# Co-expression network analyses identify functional modules associated with development and stress response in *Gossypium arboreum*

**DOI:** 10.1038/srep38436

**Published:** 2016-12-06

**Authors:** Qi You, Liwei Zhang, Xin Yi, Kang Zhang, Dongxia Yao, Xueyan Zhang, Qianhua Wang, Xinhua Zhao, Yi Ling, Wenying Xu, Fuguang Li, Zhen Su

**Affiliations:** 1 State Key Laboratory of Plant Physiology and Biochemistry, College of Biological Sciences, China Agricultural University, Beijing 100193, China; 2 State Key Laboratory of Cotton Biology, Institute of Cotton Research, Chinese Academy of Agriculture Sciences (CAAS), Anyang, Henan 455000, China

## Abstract

Cotton is an economically important crop, essential for the agriculture and textile industries. Through integrating transcriptomic data, we discovered that multi-dimensional co-expression network analysis was powerful for predicting cotton gene functions and functional modules. Here, the recently available transcriptomic data on *Gossypium arboreum*, including data on multiple growth stages of tissues and stress treatment samples were applied to construct a co-expression network exploring multi-dimensional expression (development and stress) through multi-layered approaches. Based on differential gene expression and network analysis, a fibre development regulatory module of the gene *GaKNL1* was found to regulate the second cell wall through repressing the activity of *REVOLUTA*, and a tissue-selective module of *GaJAZ1a* was examined in response to water stress. Moreover, comparative genomics analysis of the *JAZ1*-related regulatory module revealed high conservation across plant species. In addition, 1155 functional modules were identified through integrating the co-expression network, module classification and function enrichment tools, which cover functions such as metabolism, stress responses, and transcriptional regulation. In the end, an online platform was built for network analysis (http://structuralbiology.cau.edu.cn/arboreum), which could help to refine the annotation of cotton gene function and establish a data mining system to identify functional genes or modules with important agronomic traits.

Cotton, an economically important crop worldwide, is essential to the agriculture and textile industries. With the release of the whole-genome sequence of *Gossypium arboreum* in *Nature Genetics* in 2014^1^, the demand for refined annotation of cotton genes on the whole-genome level becomes high because we know so little about what most cotton genes do, such as the functional genes for fibre development, quality improvement, disease resistance, drought resistance and salinity resistance. Considering the low proportion of annotated genes in the cotton genome, it is necessary and urgent to conduct big data mining to yield novel insights into cotton development and stress response.

Currently, there are lots of transcriptomic data on *G. arboreum* available, including data on multiple growth stage tissues and stress treatment samples. High-quality genome-wide transcriptomic data sets from public databases promise to provide important biological knowledge^2^. Transcription regulation plays an essential role in establishing the gene expression profiling associated with plant development and stress response. Up to January of 2015, there were 23 RNA-seq data sets on *G. arboreum* collected in the NCBI GEO database, which include tissues such as seedling, leaf, seed and fibre. In addition, our previous experiments obtained a series of RNA-seq data for *G. arboreum* tissues in response to selective water stresses^3^. It is very time consuming to extract information from large data sets, and thus in silico methods are widely used for assistance in elucidating and annotating cotton gene functions.

Transcriptomic profiling technologies such as microarray and high-throughput sequencing (RNA-seq) enable functional association based on co-expression networks^4^. Co-expression analyses consider all samples together and establish connections among genes based on the collective information available^2^. Compared to functional studies of single genes, gene regulatory network research offers advantages such as comprehensiveness (multiple, independent data sources) and adaptation to omics data. Moreover, high conservation of transcriptional regulation among different species enables improvement of homologous gene function prediction by co-expression network analysis of cross-species. Therefore, co-expression networks can mimic gene regulatory mechanisms *in vivo* and allow modularized analysis of biological processes to discover regulatory genes or modules of vital traits.

In this study, we attempt to use the accumulated transcriptome data for *G. arboreum* gene expression analysis and develop a whole-genome-wide co-expression network with a gene expression view for biological progress analysis by combining algorithms. We seek to identify functional genes or modules in the regulatory mechanisms of important agronomic traits, especially growth and stress response. Through differential gene expression and co-expression network analyses, we identified functional modules associated with development and stress response in *G. arboreum*. For example, one regulatory module with the gene *GaKNL1* for fibre development and a water stress response module with *GaJAZ1a* were identified based on the co-expression network with gene expression view, which presents the advantages of co-expression network prediction in *G. arboreum*. In addition, the Clique Percolation Method (CPM), an approach based on analysing the overlapping community structure of networks^5^,^6^ has been used to predict possible biological processes. As a result, 1155 functional modules were identified through function enrichment tools, covering functions such as metabolism, stress responses, and transcriptional regulation. In the end, an online platform was built for the *G. arboreum* co-expression network search and gene expression analysis. In addition to the network, the online platform integrates several analysis tools for gene function prediction, such as cis-element analysis and gene ontology analysis.

## Results

### Data resources and network construction

We collected 32 expression profiling data sets on *G. arboreum* from the literature, including tissue-specific (seed, seedling, fibre, root, stem, and leaf) and stress-related (dehydration and salinity) samples, covering most growth stages of cotton (Figure S1A). This variety is beneficial for the study of growth and development regulation in *G. arboreum*. The details of these RNA-seq data are listed in Table 1. An integrated strategy was used for network construction.

First, quality control was performed to filter out low-quality reads before genome mapping by Tophat. Here, 12 RNA-seq samples of seeds from 10 dpa to 40 dpa were filtered to remove low-quality reads (quality control is described in the methods section). The quality of the remaining samples is acceptable according to the read score distribution boxplot (Figure S1B). Compared to other mapping ratios of RNA-seq samples, the ratios of 3 seedling samples (Seeds_germinated sample) were too low, and thus we ultimately retained 29 RNA-seq samples for co-expression network construction (details of mapping results are in Supplementary Table 1).

Second, gene expression values were counted and normalized to identify the lowest expression values. Here, we ran cufflinks to calculate the Fragments Per Kilobase of transcript per Million mapped reads (FPKMs) of ~41,000 genes in *G. arboreum*. To determine the minimum threshold FPKM among 29 cotton samples, we globally considered all genes’ FPKM values in each sample and finally selected a FPKM value of 0.24 as a cutoff to identify whether the gene has been expressed (Figure S1C, details are described in the Methods section). We classified the genes into two groups—the tissue-preferentially expressed group and the stress-differentially expressed group–based their FPKM values and the fold changes of the values (differential expression gene analysis is described in the Methods section).

Third, the correlation coefficients were computed to construct the co-expression network. Here, we used the Pearson correlation coefficient (PCC) to measure the co-expression relationship between two genes. According to the PCC distribution diagram of all gene pairs, the lowest 5% PCC (−0.45) and highest 5% PCC (0.65) values were considered as thresholds for negative and positive correlation (Figure S1D). To further increase the credibility of the co-expression network, we set strict parameters to filter out poor co-expression gene pairs. As a similar method was successfully used in several types of plant such as *Arabidopsis*, we followed the steps of the Mutual Rank method and classified co-expressed gene pairs into three levels: MR top3, MR ≤ 5 and 5 < MR ≤ 30 (MR is described in the Methods section). We extracted GO terms to assess co-expression networks with different cut-off values of PCC and MR. As a result, the ROC covers of co-expression network with 0.65 cut-off and MR top3 + MR ≤ 30 cut-off were better (Figure S6A,B). Finally, there are 338750 and 208018 pairs with 33413 nodes in the positive co-expression network (PCC value > 0) and negative co-expression network (PCC value < 0), respectively (Figure S1E).

Fourth, tissue-preferentially expressed genes and stress-differentially expressed genes were classified by overlaying the gene expression view onto the co-expression network. The classification rule was based on the gene expression value and fold change between the treatment and wild-type (WT) samples (rule details are described in the Methods section).

Additional, following the similar procedure of global MR co-expression network, we also constructed the tissue-specific co-expression network by 23 samples without stress treatment, and stress-treatment co-expression network by the 9 samples associated with PEG and NaCl treatment. ROC corves of tissue-specific network (Figure S6C) and stress-treatment network (Figure S6D) showed the similar tendency as well.

### Multi-dimensional network analysis

**Figure 1:**
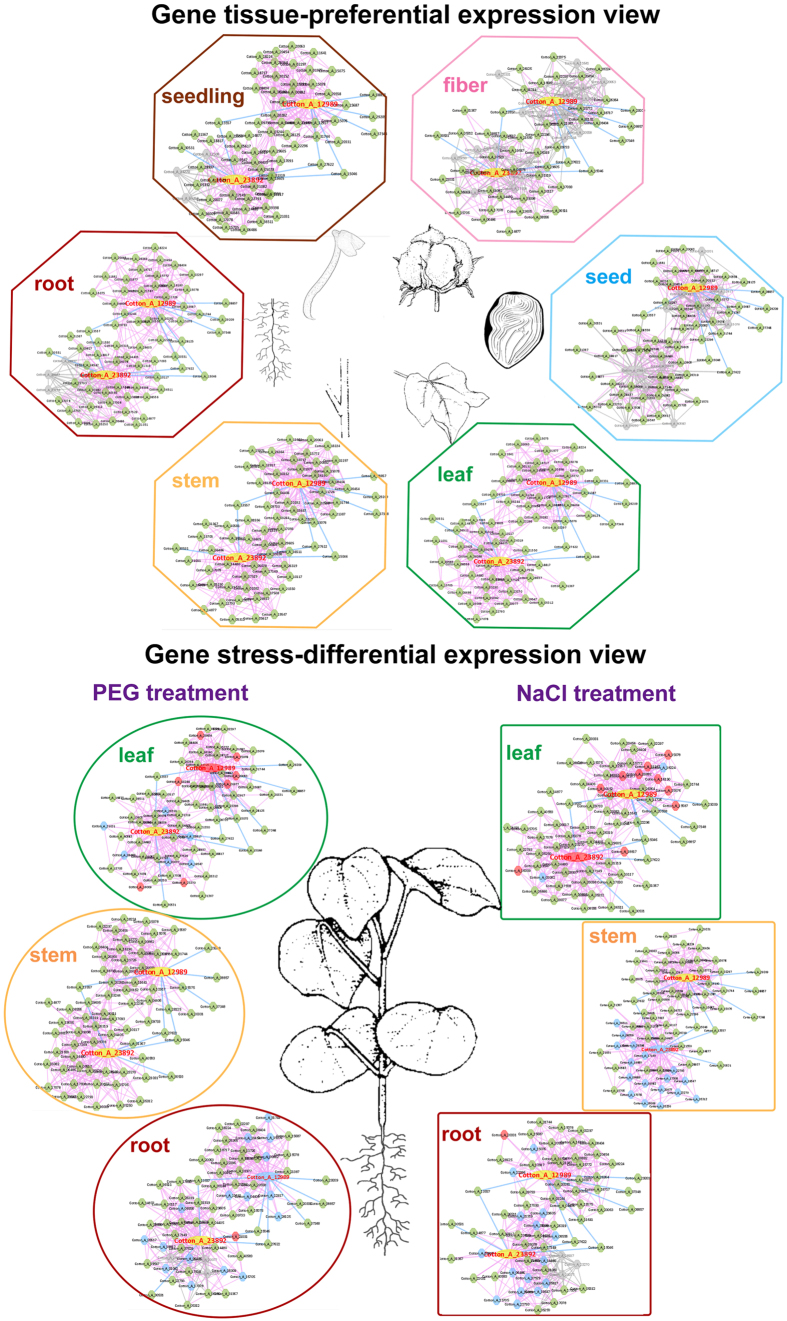
Gene tissue-preferential and stress-differential views in the co-expression network. Cotton_A_12989 (orthologue of *MYB46*) and Cotton_A_23892 (orthologue of *VND6*) were used to present regulatory modules among different growth stages and stress treatments. In “Gene tissue-preferential expression view”, there are six sub-networks of the two genes in different development stages, including seedling, root, stem, leaf, seed and fibre. The yellow nodes with red text are Cotton_A_12989 (orthologue of *MYB46*) and Cotton_A_23892 (orthologue of *VND6*). Grey and green nodes represent un-expressed and expressed genes, respectively, in the tissues. In addition, pink or blue lines represent positive or negative co-expression relationships, respectively, with Cotton_A_12989 (orthologue of *MYB46*) and Cotton_A_23892 (orthologue of *VND6*). In the “Gene stress-differential expression view”, there are two stress treatments (PEG and salt) and three tissues (root, stem, and leaf). The three sub-networks after PEG treatment are highlighted by circles, while the three sub-networks after salt treatment are highlighted by rectangles. Cotton_A_12989 (orthologue of *MYB46*) and Cotton_A_23892 (orthologue of *VND6*) are highlighted similarly in the “Gene tissue-preferential expression view”. Grey nodes represent un-expressed genes in the tissues; a red node indicates up-regulated gene expression after the stress treatment, while a blue node indicates down-regulated gene expression after the stress treatment; a green node indicates a gene without significant differences in expression level. In addition, pink or blue lines represent positive or negative co-expression relationships, respectively, with Cotton_A_12989 (orthologue of *MYB46*) and Cotton_A_23892 (orthologue of *VND6*). The pictures of growth stages are from the cotton chapter in the book *Growth Stages of Mono and Dicotyledonous Plants*.

Transcription factors (TFs) such as VNDs, SNDs, NSTs and MYBs are vital factors in the second cell wall (SCW) formation process, including the deposition of hemicellulose, cellulose and lignin, which plays important roles in plant development and in the biomass energy industry^7^,^8^. Moreover, recent studies have shown that in *Arabidopsis*, the core transcription factors *VND7* and *MYB46* participate in the stress response to iron deprivation and in ectopic xylem cell differentiation^9^. In cotton, the R2R3-type MYB transcription factor *GbMYB5* (the same type as *MYB46*) is involved in the plant adaptive response to drought stress^10^. Thus, we selected Cotton_A_12989 (orthologous gene of *MYB46,* blast result: AT5G12870, e-value: 1E-49) and Cotton_A_23892 (orthologous gene of *VND6,* blast result: AT5G62380, e-value: 2E-67) to study regulatory modules in different growth stages and stress treatments. The Z-score test showed that these genes were highly expressed in the stem compared to other tissues, and the platform constructed sub-networks with a gene expression view in six tissues for the two factors. Meanwhile, obvious differences existed among the six sub-networks: all co-expressed genes were expressed in the stem and leaf, several co-expressed genes were not expressed in the seedling or root, and many co-expressed genes were not expressed in the seed or fibre. In particular, Cotton_A_23892 was not expressed in the seed. Thus, Cotton_A_12989 and Cotton_A_23892, together with their co-expressed genes, were preferentially expressed in tissues with a more active SCW process, such as the stem and leaf. This result suggested that the tissue-preferential network could be useful for comparing differences in the modulating mechanisms among development stages (shown in the “tissue preferential analysis” part of Fig. 1). In addition to discovering changes at one level (tissue), the stress differential view displayed gene expression changes at both the tissue and stress levels. Here we showed six sub-networks with gene expression views of Cotton_A_12989 (*MYB46*) and Cotton_A_23892 (*VND6*) in the root, stem and leaf after high-osmotic pressure or high-salinity treatment. The changes resulting from PEG and NaCl treatment displayed great differences in the sub-networks. For example, there were no differentially expressed genes (DEGs) in the stem after PEG treatment, while Cotton_A_23892 (*VND6*) and several co-expressed genes were down-regulated in the stem after salt treatment. After NaCl stress treatment, Cotton_A_23892 (*VND6*) and several co-expressed genes were up-regulated in the leaf but down-regulated in the stem, while in the root, several co-expressed genes were down-regulated (shown in the “stress response analysis” part of Fig. 1). These results implied a tissue-selective effect of the stress response, as the regulatory module was more strongly affected by NaCl than by PEG in the stem. However, several genes with positive correlations overlayed non-differentially expressed in the tissue after stress treatment. For example, there are 40 genes in the MR network of Cotton_A_23892 and 4 genes are negative co-expressed with Cotton_A_23892. After PEG treatment in leaf, 27 genes are down-regulated (6 genes are significant down-regulated) and 4 of the negative co-expressed genes are up-regulated (no significant). Thus the expression view can clear present the tendency of gene differential expression in a module.

Therefore, the co-expression network with multi-dimensional analysis supplies a visualization method for analysing regulatory mechanisms in cotton development and stress response.

### Functional module identification

#### Differential gene expression analyses among developmental stages

**Figure 2:**
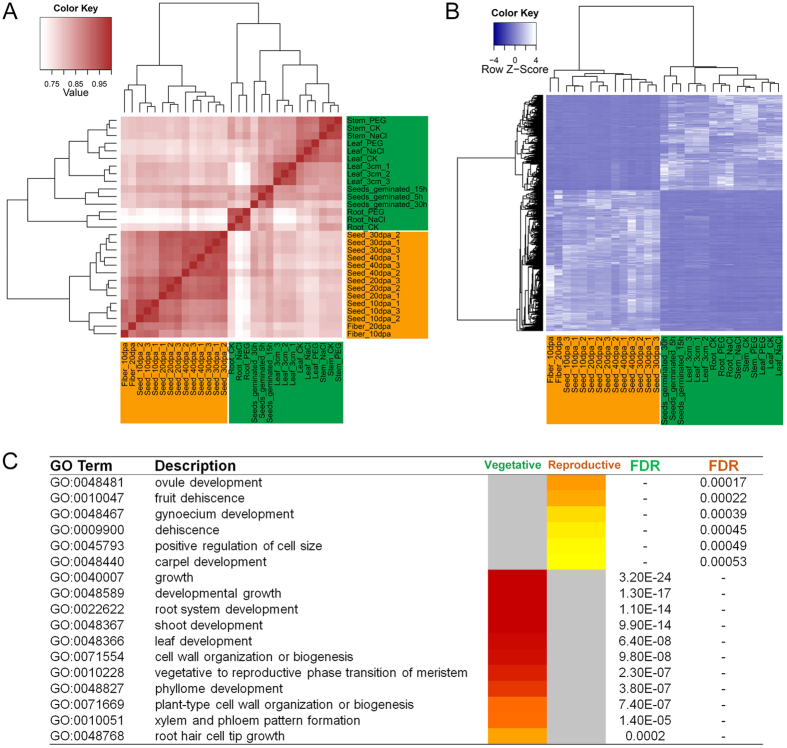
DEGs between vegetative (green) organs and reproductive (mature) organs. (**A**) The heatmap describes the clustering results for the 29 RNA-seq samples. The heatmap classifies the 29 samples into two significant groups. One group includes seedling, root, leaf and stem tissues, while the other includes seed and fibre tissues. The tissues highlighted in green are considered vegetative organs, while tissues highlighted in orange are considered reproductive organs. (**B**) The gene expression profiling heatmap clusters 1752 DEGs into two groups, vegetative (green) and reproductive (orange). Genes are clustered vertically, and tissues are clustered horizontally. The DEGs of vegetative oranges are highly expressed (light blue or white) in the green-coloured tissues and lowly expressed (dark blue) in the orange-coloured tissues. The DEGs of reproductive oranges are highly expressed (light blue or white) in the orange-coloured tissues and lowly expressed (dark blue) in the green-coloured tissues. (**C**) Custom GO enrichment analyses of 1752 DEGs were performed by agriGO, maintaining a FDR < 0.05. The bars are coloured from dark red to light yellow from the most significantly enriched GO entry to the least significant GO entry, respectively. A grey-coloured bar indicates a GO entry without significant differences in vegetative or reproductive tissues. Tissue-specific GO entries are listed in this table; for example, genes highly expressed in vegetative organs (green) function in growth and development, while genes highly expressed in reproductive organs (mature) function in ovule, fruit and gynoecium development.

To examine regulatory mechanisms at a single developmental stage or the transitions between different growth stages, we analysed the gene expression profiles among different tissues to detect important functional modules with co-expression networks. Correlation is frequently used as an evaluative criterion to describe the relationships among next-generation sequencing data, and thus the Spearman Rank Correlation Test was used to classify stages of the 29 RNA-seq samples. It is clear that the tissues belong to two categories: vegetative organs, including the seedling, root, leaf and stem; and reproductive organs, including the different seed stages and fibre (Fig. 2A)^11^. As DEGs may be involved in the transition between developmental stages^11^, key regulators or regulatory modules of cotton growth, such as those for fibre development, could be found by DEG analysis. Methods such as the t-test and fold change of gene FPKM values were used to find DEGs. A total of 1752 DEGs were found between the vegetative and reproductive organs, more than half of which were up-regulated in the reproductive organs (Fig. 2B, Supplementary Dataset S1). Custom gene ontology (GO) analysis was performed on the DEGs that were highly expressed in either the vegetative or reproductive organs to define their functions. It is meaningful that the highly expressed genes in vegetative organs function in growth and development, while the highly expressed genes in reproductive organs function in ovule, fruit and gynoecium development (Fig. 2C). Therefore, these DEGs and their co-expressed genes may play important roles in the cotton growth cycle.

TFs control the expression of their targets by binding to conserved cis-elements to perform their regulatory functions. As a result, TFs may share similar or opposite expression profiling with their targets in the case of positive regulation or negative regulation, respectively. As a co-expression network clusters genes with highly positive or negative relationships caused by co-regulation in the same pathway, it can predict functional modules effectively and mimic regulatory mechanisms.

**Figure 3:**
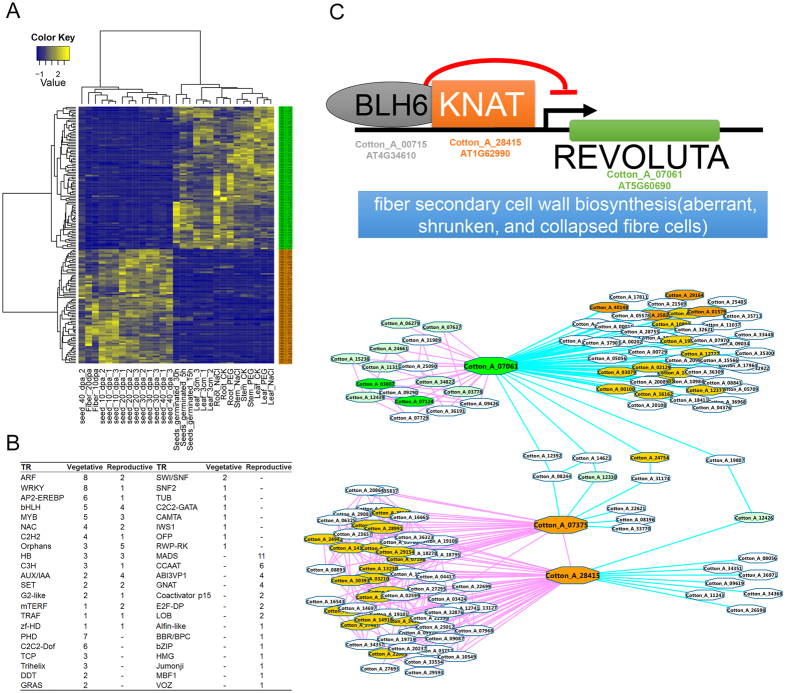
Fibre synthesis regulatory module in transitions from vegetative to reproductive growth. (**A**) The gene expression profiling heatmap clusters 162 differentially expressed TFs into two groups, vegetative (green) and reproductive (orange). Genes are clustered vertically, and tissues are clustered horizontally. A yellow-coloured box represents a gene highly expressed in a tissue, and a dark blue box represents a gene lowly expressed in a tissue. The gene IDs highlighted in green are highly expressed in vegetative tissues, and gene IDs highlighted in orange are highly expressed in reproductive tissues. (**B**) The table lists the distribution of 162 differentially expressed TFs between the vegetative and reproductive groups. (**C**) The module describes how *KNAT7* regulates fibre and SCW development through binding to the promoter of *REV* and repressing its transcriptional activity. The co-expression network download clearly displays the repression of Cotton_A_07061 (*REV*) by the regulation of Cotton_A_28415 (*KNAT7*) from the vegetative stage to the reproductive stage. A pink line between two nodes indicates a positive co-expression relationship, while a blue line between two nodes indicates a negative co-expression relationship. A green (dark or light green) node indicates a gene highly expressed in vegetative tissues, and an orange (dark or light orange) node indicates a gene highly expressed in reproductive tissues. In addition, the dark green and orange nodes belong to the 162 differential expressed TFs.

There are 162 transcription regulators among the 1752 DEGs that may have potential roles in regulating cotton growth and maturation (Fig. 3A,B, Supplementary Dataset S1). For example, Cotton_A_28415, a class II KNOX transcription factor, is orthologous to cotton KNL1 (*GhKNL1*), which participates in fibre development by modulating the expression of the genes related to cell elongation and secondary wall biosynthesis of cotton fibres. Furthermore, *GhKNL1* partially rescues the defective phenotype of the *Arabidopsis knat7* mutant, which means that *GhKNL1* and KNOTTED ARABIDOPSIS THALIANA7 (*KNAT7*) should be homologous^12^. Furthermore, Liu *et al*. have discovered that *KNAT7* can interact with BEL1-LIKE HOMEDOMAIN6 (*BLH6*) to repress the activity of REVOLUTA/INTERFASCICULAR FIBERLESS1 (*REV/IFL1*) through binding to its promoter region and thus regulates SCW formation in *Arabidopsis*^13^ (Fig. 3C). According to orthologue analysis, Cotton_A_07061 is identified as REV in *G. arboreum* and belongs to the 162 TFs among the DEGs (Fig. 3B). It is quite exciting that the expression profiles of two transcription factors (Cotton_A_07061 and Cotton_A_28415) are opposite among the 29 samples as well as in *Arabidopsis*: Cotton_A_07061 (*REVOLUTA*) is highly expressed in the vegetative organs, while Cotton_A_28415 (*KNAT7*) is highly expressed in the reproductive organs (Fig. 3A,C). In addition, the co-expression network shows that in the sub-network of Cotton_A_07061 (*REVOLUTA*), the positively co-expressed genes are generally highly expressed in vegetative tissues, whereas the negatively co-expressed genes are generally highly expressed in the reproductive tissues. For example, Cotton_A_07124, an orthologue of *VND4* in *Arabidopsis*, is expressed in the developing xylem and regulates SCW growth^14^; whereas the MADS-box family Cotton_A_40148, the orthologue of *AGAMOUS*, is specifically expressed in mature parts such as the floral meristem, carpel and stamen^15^. The genes positively and negatively co-expressed with Cotton_A_28415 (*KNAT7*) display the reverse tendency. Therefore, the co-expression network can imitate the potential regulatory mechanism of growth stage transitions in cotton. Conserved motifs have been scanned among 3 kb promoter sequences of the co-expressed genes. A large number of similar DNA sequences with a TGAC core motif, which is bound by BLH and KNOX proteins, have been observed in those promoters^13^. In addition, transcription regulator binding sites appear in the 3 kb promoters. For example, the BS1INAG motif with the conserved sequence “AAATTAAA” appears at more than 500 sites and participates in the repression of AGAMOUS by BELLRINGER in floral and inflorescence meristems^16^. Other conserved motifs are listed in Supplementary Dataset S1.

In addition to displaying known regulatory mechanisms, the co-expression network can predict potential functional modules. For example, Cotton_A_07375, one of the G2-like transcription regulators, shows a similar co-expression network to Cotton_A_28415 (*KNAT7*) with Cotton_A_07061 (*REVOLUTA*), which suggests that Cotton_A_07375 may be a candidate gene for the regulation of fibre development.

#### Differential gene expression among stress-treatment conditions

In severely dry and hot climates, *G. arboreum* shows good genetic stability and important stress tolerance properties. Previous studies have found possible mechanisms related to the cotton water-stress response, but the possible regulatory pathways involved in water stress in cotton are not well understood^17^,^18^.

Thus, research on the water stress response mechanism in *G. arboreum* is important for the improvement of the germplasm. Excitingly, we previously generated 9 RNA-seq samples, including root, stem, leaf tissues with PEG and salt treatments. These multi-dimensional data^3^ provide an opportunity to analyse DEGs in different tissues after stress treatments.

**Figure 4:**
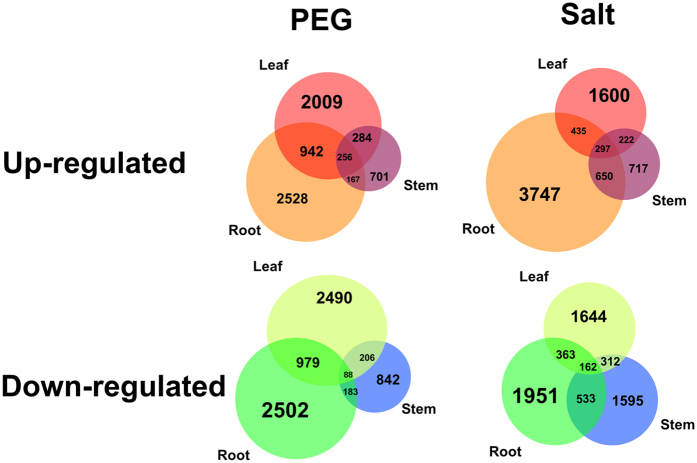
Venn diagrams describing gene expression variation among three tissues after PEG and salt treatment. The Venn diagram in the upper left corner displays overlaps among up-regulated genes in response to PEG treatment in the root (orange), leaf (red) and stem (purple). The Venn diagram in the upper right corner displays overlaps among up-regulated genes in response to salt treatment in the root (orange), leaf (red) and stem (purple). The Venn diagram in the lower left corner displays overlaps among down-regulated genes in response to PEG treatment in the root (dark green), leaf (fluorescent green) and stem (blue). The Venn diagram in the lower right corner displays overlaps among down-regulated genes in response to salt treatment in the root (dark green), leaf (fluorescent green) and stem (blue).

Venn diagram analysis clearly describes the similarities and differences in gene expression changes in response to different treatments among different tissues (Fig. 4). After PEG or salt treatment, the variation of gene expression presents distinct trends in all three tissues, and the overlapping genes represent a fraction of all the altered genes. Notably, the largest number of DEGs occur in the root after both stress treatments, which could be because the root directly accesses the growth medium with PEG or salt, while the leaf and stem are affected indirectly. In response to PEG treatment, there are more altered genes in the leaf and root than the stem, but the leaf and stem have more altered genes in common. As PEG can cause cell dehydration, the drought signal is transferred from the root and therefore forms an increasing gradient of osmotic pressure from the root to the stem and then leaf. In addition, the opening and closing of stomata and changing phytohormone levels in the leaf play roles in response to water loss, which could result in more altered genes in the leaf than the stem. In the salt treatment condition, there are significantly more up-regulated genes in the root, twice as many as the number of up-regulated genes in the leaf and three times more than the number of up-regulated genes in the stem. However, the number of down-regulated genes is similar in all three tissues. This result is likely because Na^+^ passes to the leaf through the stem after absorption by the root, and a decreasing ionic concentration gradient exists from the root to the stem and leaf. Apart from the poisoning effect of sodium, a high ionic concentration could decrease the water potential, also leading to a water deficit. Thus, the higher number of DEGs in the root after salt treatment may be due to multiple effects, such as both drought and salinity.

**Figure 5:**
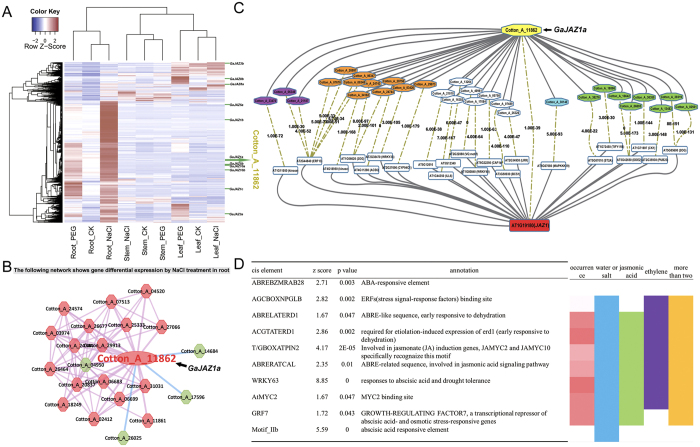
A regulatory module for salinity in root. (**A**) Gene expression profiles of 5129 up-regulated genes after salt treatment in root samples cluster in 3 tissue samples with 2 stress treatments. Genes are clustered vertically, and tissues are clustered horizontally. A red box represents a gene highly expressed in a sample; a white box represents a gene without significant expression changes; a blue box represents a gene lowly expressed in a sample. A majority of these genes were more active in the Root_NaCl sample. In addition, the JAZ family, highlighted by green lines at the right, displays high enrichment in the gene set. (**B**) The sub-network with a gene expression view of Cotton_A_11862 (*GaJAZ1a*) displays DEGs in the root after salt treatment. A pink line between two nodes indicates a positive co-expression relationship, and a blue line between two nodes indicates a negative co-expression relationship. A green node indicates a gene without significant differential expression in the root after salt treatment. A red node indicates up-regulated expression after stress treatment. (**C**) Comparison of co-expression networks between Cotton_A_11862 (*GaJAZ1a*) in *G. arboreum* and AT1G19180 (*JAZ1*) in *Arabidopsis*. Dotted lines link orthologous pairs between the two species, and the numbers in the middle of the dotted lines are the e-values of the BLAST results. Based on GO analysis, the cotton genes of the four functional groups, including water or salt stress, jasmonic acid-related pathways, ethylene-related pathways and functions involved in more than two pathways are highlighted in blue, green, purple and orange, respectively. (**D**) The table lists key cis-elements involved in stress response signalling pathways. The red bar on the right represents the frequency of co-expression genes containing the motif. The darker the red colour, the more co-expressed genes contain this cis-element. Other coloured bars, including blue, green, purple and orange bars, represent the four pathways. For example, the motif AGCBOXNPGLB has a significant frequency of occurrence in the genes of the “water or salt stress”, “ethylene-related pathways” and “more than two pathways” groups but is absent in the genes of “jasmonic acid-related pathways”.

According to the Venn diagram, the root is more sensitive to salt stress than other tissues. To study the regulatory mechanism of resistance to salinity, we selected 5,129 up-regulated genes in the root after salinity treatment. Heatmap analysis of gene expression profiling shows that the expression values of most of these genes increased significantly in the salt-treated root sample (Fig. 5A). JASMONATE-ZIM-DOMAIN (JAZ), a small family in *Arabidopsis,* was reported to dynamically regulate jasmonate (JA) signalling pathways to regulate activities such as signal transduction in plant stress response, growth and development^19^,^20^, including stresses such as freezing^21^, wounds and shade^22^. Recently, a non-TIFY JAZ repressor, *JAZ13* (encoded by At3g22275), was discovered and is most closely related to *JAZ8*, which includes divergent EAR, TIFY/ZIM, and Jas motifs^23^. Based on the multiple sequence alignment and phylogenetic tree, there are seven JAZ family members with 14 genes in *G. arboreum* (Figure S2). Interestingly, many JAZ family members displayed significantly up-regulated expression in the salt-treated root sample (Fig. 3A). Four JAZ family genes, *GaJAZ1a, GaJAZ1b, GaJAZ3a*, and *GaJAZ5b*, were selected for Q-RT-PCR to validate the mRNA-seq results in nine samples, including the root, stem, and leaf tissues under PEG and NaCl treatments and a control condition. We performed one-by-one comparisons of each transcript between Q-RT-PCR and mRNA-seq results. The Q-RT-PCR results matched the mRNA-seq expression patterns very well (Figure S3). A recent report suggested that *GhJAZ1* could modulate the plant defence-to-development transition in *Gossypium hirsutum* through the transactivation of *GbWRKY1 (Gossypium barbadense*)^24^. Therefore, the appearance of Cotton_A_11862 (*GaJAZ1a*), an orthologue of *GhJAZ1,* in *G. arboreum* may indicate a role in regulating salt stress tolerance. According to the stress-differential co-expression network analysis of Cotton_A_11862 (*GaJAZ1a*), a majority of the co-expressed genes were more active after salt treatment in the root, which means these genes may participate in a signalling pathway in response to salt stress (Fig. 5B). As the cis-element is a vital mechanism at the transcriptional level, cis-element significance analysis was performed on 3 kb of the co-expressed genes’ promoter regions. Several key cis-elements were found to be significantly enriched. For example, the motif T/GBOXATPIN2 occurs in JA-induced genes and is specifically recognized by *JAMYC2* and *JAMYC10*^25^, and the motif ABRELATERD1 is required for early response to dehydration stress^26^. Furthermore, the co-expressed genes with these cis-elements exhibit functions such as response to water or salt stress and participate in phytohormone signalling pathways. The results above show that the functional module consisting of *GaJAZ1a* and its co-expressed genes may regulate the cotton response to salt stress through phytohormone signalling pathways such as abscisic acid (ABA), JA and ethylene pathways (Fig. 5D). The model plant *Arabidopsis* has a close evolutionary relationship with cotton, and therefore, comparative genomics could help to construct and identify functional modules in *G. arboreum*. Here, the top 300 PCC co-expressed genes of *Arabidopsis* were collected from ATTED-II and we also selected the top 300 PCC co-expressed gene of *G. arboreum* to make comparison. The co-expression networks of Cotton_A_11862 (*GaJAZ1a*) and AT1G19180 (*JAZ1*) reveal high similarities, such as orthologue gene pairs in each network, which help to increase the credibility of predicting candidate regulators and functional modules. In addition, the GO enrichment analysis of these co-expressed genes indicates that biological processes such as “hormone signalling pathway”, “response to salt stress”, “response to water deprivation” and “abscisic acid-mediated signalling pathway” are enriched. Thus, we classified these genes into four functional groups, namely water or salt stress, jasmonic acid-related pathways, ethylene-related pathways and functions involved in more than two groups. Finally, the cis-element and GO enrichment analyses were considered together, and 30 co-expressed genes together with Cotton_A_11862 (*GaJAZ1a*) were considered as candidates to modulate water stress and salt tolerance in cotton (Fig. 5C, Supplementary Dataset S2).

#### Functional module identification on the whole-genome level

The Clique Percolation Method (CPM)^5^ was used to find clusters or modules with nodes more densely connected to each other than to nodes outside the group in the cotton co-expression networks. Parameter selection was based on more modules, more gene coverage and more community overlap. Here, we selected a k = 6 clique size, which means each node had co-expression interactions with at least six nodes in a module (Figure S4). The functions of modules were predicted through integrating annotations such as GO, gene families (transcription regulators, kinase, and carbohydrate-active enzymes), and Kyoto Encyclopaedia of Genes and Genomes (KEGG), and non-significant entries were filtered by the Fisher test and multiple hypothesis test (false discovery rate (FDR) ≤ 0.05). In the end, 1,155 modules containing 6 to 99 genes each were found in *G. arboreum*, covering functions such as metabolism, pathogen and stress responses, hormones, development, and transcriptional regulation (Supplementary Dataset S3). The connections between functional modules could represent crosslinks among different pathways *in vivo*, and thus modules with three nodes connected to other modules were selected. As a result, 493 functional modules in *G. arboreum* showed relationships with other modules.

### Salt tolerance analysis of transgenic *Arabidopsis* over-expressing the *GaJAZ1a-like* gene

**Figure 6:**
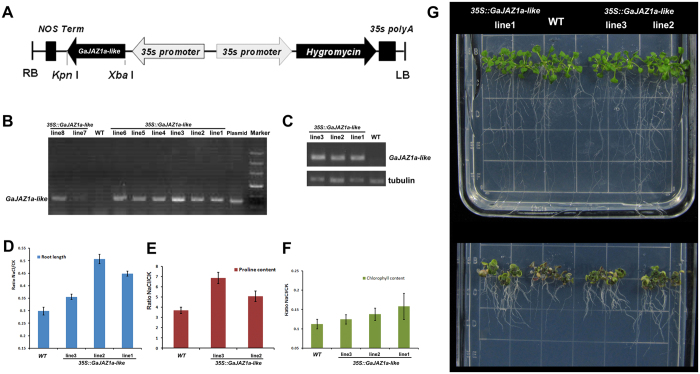
Phenotype of transgenic *Arabidopsis* over-expressing the *GaJAZ1a-like* gene. (**A**) The binary vector super-1300 construct of *GaJAZ1a-like* was used for transgenic *Arabidopsis* transformation. (**B**) PCR analysis of *GaJAZ1a-like* in Arabidopsis *35S*::*GaJAZ1a-like* transgenic lines. (**C**) RT-PCR of *GaJAZ1a-like* expression results in Arabidopsis *35S*::*GaJAZ1a-like* transgenic lines. (**D**) Length of transgenic and WT Arabidopsis plant roots under 150 mM NaCl treatment. (**E**) Free proline contents of WT plants and *35S*::*GaJAZ1a-like* transgenic Arabidopsis plants under NaCl treatment. (**F**) Percentage of chlorophyll relative content of WT plants and *35S*::*GaJAZ1a-like* transgenic Arabidopsis plants under NaCl treatment. (**G**) Comparison of three *35S*::*GaJAZ1a-like* Arabidopsis transgenic lines (line1, line2, and line3) with the WT under 150mM NaCl treatment.

Members of the JAZ family were reported to play important roles in JA signal transduction, and a large number of cotton JAZ homologues were up-regulated during salt stress, especially in the root tissue (Fig. 5A). To clarify the physiological roles of cotton JAZ in salt stress response in plants, we cloned the full-length cDNA for one cotton JAZ1, *GaJAZ1a-like* (based on the PlantGDB EST assembly PUT-157a-Gossypium-34778) and applied a transgenic approach to study its potential function in the salt response. We generated transgenic *Arabidopsis* over-expressing the *GaJAZ1a-like* gene under the control of the 35S promoter (Fig. 6A). Eight independent hygromycin-resistant transgenic *Arabidopsis* lines were generated and confirmed by PCR (Fig. 6B). The expression levels of *GaJAZ1a-like* in selected T2 transgenic plants were analysed by RT-PCR (Fig. 6C). Further phenotypic analyses were performed on the lines with higher expression levels of *GaJAZ1a-like*. After germination, seedlings from each line (including WT and transgenic *Arabidopsis* lines) were carefully transferred to new Murashige&Skoog (MS) medium containing 150 mM NaCl for treatment. Differences in the 2-week growth were observed in the different treatment media (Fig. 6G). There were no visible morphological changes between the WT and transgenic lines over-expressing *GaJAZ1a-like* in the MS medium. However, in the150 mM NaCl medium, the growth of the control plants was strongly inhibited, as the plants displayed short roots and brown leaves, whereas *35S*::*GaJAZ1a-like* transgenic seedlings showed a salt-tolerant phenotype compared to the WT plants, with longer roots and more green leaves. We measured the root length (Fig. 6D), proline content (Fig. 6E), and chlorophyll content (Fig. 6F) in these plants with and without salt treatment. After salt treatment, the root lengths of the transgenic lines were more than 20% longer than in the WT, and the difference was highly significant (P < 0.01). The difference in proline content before and after salt treatment was significantly higher in the transgenic lines than in the WT plants (P < 0.05). The transgenic lines also retained a more appropriate chlorophyll proportion than the WT plants after salt treatment.

### Online co-expression network database for *G. arboreum*

Here, a co-expression network search function for a single gene or a list of genes is provided. Different categories of the co-expression network can be selected to visualize sub-networks, such as a single positive co-expression sub-network, a single negative co-expression sub-network or a sub-network with two co-expression relationships. The network is visualized using the Cytoscape web tool, and a sub-network could be rebuilt and displayed more clearly through dragging interested nodes (Figure S1G). Notably, there are two main analysis options in the co-expression network platform: the tissue-preferential expression view and the stress-differential expression view. In the tissue-preferential analysis, six growth stage tissues of *G. arboreum* are included, namely the seedling, root, stem, leaf, seed and fibre. Instead of presenting a common co-expression network among all growth stages, the tissue-preferential expression view displays a series of regulatory modules from vegetative development to reproductive development, mimicking the dynamic changes and highlighting the differences in regulatory modules during growth (Fig. 1). After selecting one of the six tissues (e.g., root), the gene expression changes of a certain sub-network can be clearly observed. Nodes and edges in grey represent genes that are not expressed in the selected tissue (such as root) (Figure S1H). In stress-differential analysis, changes on two levels (both tissue and stress) in a sub-network are presented (Fig. 1). The stress-differential analysis displays not only the differences in gene expression among tissues but also the fold changes of gene expression after stress treatment. Here, the nodes in red represent up-regulated genes, while nodes in blue represent down-regulated genes in a tissue after a stress treatment (e.g., high salinity in the leaf) (Figure S1I). All co-expressed gene pairs in the sub-network are listed with detailed information, including PCC scores, MR scores and co-expression relationships (Figure S1K). All genes in the sub-network are listed with annotations, including their orthologues and functional annotation in *Arabidopsis* (Figure S1J), along with the fold changes of DEGs (Figure S1L).

The tissue-specific co-expression network and stress-treatment co-expression network (construction details in methods) are also provided to make comparisons with global co-expression network (Figure S7A). Similarities and diversities are clearly presented in different kinds of co-expression networks: the ratio of overlaps between tissue-specific and global co-expression networks is higher, while the ratio of overlaps between stress-treatment and global co-expression networks is lower (Figure S7B-C, S8B-C). The details of comparison result, like overlaps or differences of nodes and edges are listed and highlighted with colors (Figure S7D–G).

As an alternative to the co-expression network search, several tools have been developed for gene annotation, such as keyword search, cis-element analysis and gene set (GSEA) enrichment analysis, which can annotate a list of genes with functions. For more information, the co-expressed genes with top 300 rank of PCC were also kept and could be browsed by clicking “Top300 PCC genelist” in the gene detail page.

Finally, we combined these results into a platform named the “*Gossypium arboreum* co-expression network” for search and visualization, including tissue-preferential and stress-differential analysis, with user-friendly tools. The website can be accessed at http://structuralbiology.cau.edu.cn/arboreum (Figure S1F).

## Discussion

### Summary of structure and function analysis in the co-expression network with a gene expression view

In this study, we used existing transcriptome data on *G. arboreum* to construct a whole-genome co-expression network, containing 80% of predicted genes and 546,768 positive and negative edges. The samples cover most of the development stages of cotton growth and maturity, such as the seedling, root, leaf, stem, seed and fibre, as well as the effects of abiotic stress treatments such as dehydration and salinization in the leaf, root and stem. Therefore, multi-dimensional samples (developmental stages and abiotic stress treatment) and a comparable measure (Rank of Correlation Coefficient)^27^ have been used to successfully build a network-based platform to refine the annotation of cotton genes or modules with functions relating to important agronomic traits, especially for growth stage transitions such as fibre elongation and stress responses such as those to water stress. Meanwhile, a data mining system has been combined with several functional analysis tools, including orthologue annotation, gene family classification, cis-element analysis and gene ontology analysis, to evaluate the reliability of the predictions.

### Superiority of overlaying gene expression profiles onto the co-expression network in *G. arboreum*

Protein-protein interaction (PPI) networks and co-expression networks are useful for gene function identification and functional module analysis. Model plants, especially *Arabidopsis,* have an associated approximately 20,000 experimental interaction pairs stored in several public databases, such as TAIR^28^, BAR^29^, DIP^30^, MINT^31^, IntAct^32^ and BioGRID^33^, greatly contributing to PPI network construction. Unlike in *Arabidopsis*, however, a lack of PPI data has blocked proteomics data mining and regulatory module discovery in cotton. Therefore, co-expression networks have become the preferred method for functional analysis. Currently, a complete genome sequence of *G. arboreum* has been published, along with a relatively comprehensive set of RNA-seq samples of varying developmental stages and stress responses, allowing the development of the co-expression network. There are fewer categories of expression profiling data than for the well-studied *Arabidopsis*; for example, reproductive tissues such as the petal are not included, and the stress treatment samples lack repetition. However, a global co-expression network with options for tissue-preferential analysis and stress-differential analysis was constructed using these relatively comprehensive samples, and the regulatory modules discovered by the network analysis were meaningful and displayed high reliability, which was confirmed by publications.

For clear visualization and convenient analysis, a platform based on the Cytoscape tool was used to display the multi-dimensional network structure. The network can be searched by a single gene or a list of genes, and pink or blue lines distinguish positive and negative co-expression relationships (Figure S1G). In addition, tissue-preferential analysis can highlight genes that are preferentially expressed in a certain tissue, while unexpressed genes are hidden (Figure S1H, Fig. 1). Moreover, stress-differential analysis is a two-dimensional (development and stress) tool, which can highlight up-regulated genes in red and down-regulated genes in blue after a given stress treatment in a particular tissue (Figure S1I, Fig. 1). For example, Fig. 5B shows that many genes (red nodes) in the *GaJAZ1* regulatory module were up-regulated in the root after salt treatment.

To make network analysis more meaningful, the tissue-specific co-expression network and stress-treatment co-expression network are provided to make comparisons with global co-expression network in the website (Figure S7A). Similarities and diversities are clearly presented in different kinds of co-expression networks, like the ratio of overlaps between tissue-specific and global co-expression networks is higher than that of stress-treatment co-expression (Figure S7B-C, S8B-C).

Thus, the platform “*Gossypium arboreum* co-expression network” was developed for the convenient search and visualization of functional regulatory module analysis.

### Strategies and improvements for functional module identification

Instead of identifying a single gene function, the co-expression network was developed to detect functional modules with multiple genes that could regulate important agronomic traits, such as the production and quality of fibre development and response to environment stress. Moreover, presenting positive and negative co-expression relationships at the same time enhances the understanding of regulatory mechanisms. For example, the Cotton_A_28415 (*KNAT7*) and Cotton_A_07061 (*REVOLUTA*) functional module illustrates a possible biological process that regulates the transition from vegetative to reproductive growth by changes in key gene expression profiles and is useful for studying fibre biosynthesis (Fig. 3C). Furthermore, multi-dimensional data with tissues and stress treatments can be used in a stress-related co-expression network for identifying abiotic stress response functional modules in *G. arboreum*. For example, stress-differential analysis clearly displayed the gene expression profile change of *GaJAZ1a* in the root, which was consistent with the literature (Fig. 5B). Moreover, the high similarities found between orthologue networks through comparative genomics have increased the credibility of predicting candidate regulators for modulating the salt tolerance of cotton (Fig. 5C). Common cis-elements could be an effective method for predicting regulators inside a module, as the shared motifs in the regulators’ promoter regions result in co-expression relationships at the transcriptional level. For example, the 30 components of the root salt-sensitive module *GaJAZ1a* are significantly enriched in the dehydration stress^26^ and phytohormone response motifs (P-value < 0.05), illustrating that co-expressed genes may regulate the response of cotton to salt stress through phytohormone signalling pathways such as the ABA, JA and ethylene pathways (Fig. 5D).

Notably, however, cis-elements with annotation in plants are too few for function prediction. Only a few databases collect these elements, such as the Plant Cis-acting Regulatory DNA Elements (PLACE) database^34^, AthaMap webserver^35^ and PlantCARE^36^. Thus, we need more epigenomics data, such as ChIP-chip, ChIP-seq and DNase-seq data, to predict possible DNA-binding sites.

Based on the multi-dimensional co-expression network, the strategy for functional module prediction and refined gene function annotation is general and effective, and thus more regulatory modules could be identified by the same strategy based on a detailed biological focus or event, such as increasing yields. Here, we piloted several algorithms that have been successfully used to predict regulatory networks in plants, such as the APCC (Average Pearson Correlation Coefficient), Bayesian Network and k-means clustering^11^, but the results were unsatisfactory owing to the lack of complete data on cotton development and mature tissues. However, the CPM method^5^ successfully identified 1,155 functional modules based on a multi-dimensional co-expression network, and the functional enrichment analysis results covered metabolism, pathogen and stress responses, hormones, development, and transcriptional regulation. Therefore, these findings contribute to understanding the molecular regulatory mechanisms of vital agronomic traits, such as high yield, high quality and stress tolerance, which can improve the molecular breeding of cotton.

As the most widely cultivated fibre resource, *G. hirsutum* has accumulated substantial expression profiling data, such as tiling arrays and RNA-seq. We have already collected the 115 public RNA-seq samples of *G. hirsutum* and calculated their FPKM values. Here, we used *KNAT7* as an example and compared the expression patterns of its orthologous genes in *G. arboreum* and *G. hirsutum*. There are two highly conserved orthologous genes in *G. hirsutum*, namely Gh_D08G1910 (*GhKNL1*) and Gh_A08G1599. The top 40 genes sharing high co-expression relationships with Gh_D08G1910 (*GhKNL1*) and Gh_A08G1599 were selected for expression profiling analysis. Based on this co-expression network, most of the genes co-expressed with Cotton_A_28415 are also highly expressed in the reproductive organs (Figure S5A). Similarly, the expression profiling heatmap of *G. hirsutum* shows that the two Knotted family genes and most of their co-expressed genes are highly expressed in reproductive tissues, especially the stamen and fibre (Figure S5B). Thus, the regulatory functional module may be conserved between *G. hirsutum* and *G. arboreum*. Since the genome of *G. hirsutum* is large and complex^37^,^38^, its regulatory module analysis is also urgent. Based on their successful use in *G. arboreum*, more comprehensive data on tissues from different development stages and stress treatment experiments will allow the construction of a multi-dimensional co-expression network in *G. hirsutum*, and the differences between tissue and stress treatment sub-networks may be more significant than in *G. arboreum*.

## Conclusion

Here, multi-dimensional samples and comparable computing measures have been used to build a co-expression network to refine the annotation of cotton genes or functional modules with important agronomic traits, such as growth stage transitions including fibre elongation and stress responses including the response to water stress. Meanwhile, module functional enrichment analysis tools, such as gene family classification, cis-element analysis and gene ontology analysis, could be used to evaluate the reliability of the predictions. Based on the gene expression analysis and conditional network, the strategy for functional module prediction and refined gene function annotation is general and effective. Thus, more regulatory modules could be identified by the same strategy based on a detailed biological focus or event, such as increasing yields. Therefore, this approach will contribute to understanding the molecular regulatory mechanisms underlying vital agronomic traits, such as high yields, high quality and stress tolerance, to improve the molecular breeding of cotton. Given the important role cotton plays in our lives, we hope that more omics data, including genomics, transcriptomics, proteomics and epigenomics data, could improve the network analysis for functional module identification and reduce biases or mistakes caused by limitations, thereby benefitting cotton breeding.

## Materials and Methods

### Data mapping and gene expression profiling analysis

The whole-genome sequencing data were accessed from the 2014 public version^1^ and corresponded to a genome size of 1.7 Gb, 13 pseudo chromosomes and 41,330 protein-coding genes. The read quality of twelve seed RNA-seq samples was poor, and therefore, we used fastx-toolkit v0.0.13 to cut 49 bp from the 5′ terminal ends of the reads and filtered out low-quality reads based on a minimum quality score, retaining 80% of bases that must have 20 quality. We then used tophat v2.0.9, bowtie2 v2.1.0 and samtools v0.1.19 to map RNA-seq reads on the genome of *G. arboreum* with default parameters. As a result, the mapping ratio increased by approximately 10% in the seed samples. All mapping ratios are listed in Supplementary Table 1. The FPKMs of each gene were computed by cufflinks v2.2.0 with default parameters.


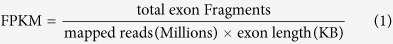


FPKM, fragments per kilobase of exon per million fragments mapped.

To determine the minimum threshold of the gene expression value (FPKM) among 29 cotton samples, the lowest 5% of all gene FPKM values in each RNA-seq sample and the standard deviation (SD) of each experimental group were computed. Then, the mathematical formula “threshold = average(5% value) + 3 * SD” was used to calculate the minimum expression value of each experimental group. The minimum threshold of FPKM was 0.24.

### Co-expression network construction algorithm and parameters

The PCC was used to link genes with high co-expression relationships among the 29 samples. Furthermore, MR was used to calculate the rank of PCC through taking a geometric average of the PCC rank from gene A to gene B and from gene B to gene A. Specifically, when gene A is the third highest co-expressed genes for gene B, PCC rank of gene A to gene B is 3. Thus, MR ensures more credible co-expression gene pairs, which are then used to construct a co-expression network.

Pearson correlation coefficient:


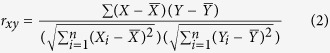


X or Y represents the FPKM values of a gene, and n represents the number of tissues (here n = 29).

MR:


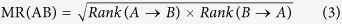


Here, we retained co-expressed gene pairs with a single direction rank of PCC (Rank_AB_ or Rank_BA_) less than 3 and MR score less than 30 (bold or normal edges) in a co-expression network^39^.

Following the similar procedure, the tissue-specific co-expression network was constructed by 23 samples without stress treatment, and stress-treatment co-expression network was constructed by the 9 samples associated with PEG and NaCl treatment.

Finally, the MR co-expression gene pairs are regarded as having positive co-expression values when their PCC values are more than zero and negative co-expression values when their values are less than zero.

### DEG analysis and the definition of tissue-preferentially and stress-differentially expressed genes

DEGs were detected by the two-tailed t-test, Benjamini-Hochberg multiple testing correction (FDR) and fold changes of gene expression values. Genes with an appropriate FDR and fold changes of FPKMs were regarded as DEGs.

In the tissue-preferential gene group, if one gene’s FPKM was greater than 0.24 in a tissue sample such as leaf, then it was displayed in the leaf sub-network; in the stress-response gene group, when the gene log2 fold change of FPKM values between treatment and WT (NaCl/CK or PEG/CK) was ≤−1 or ≥1, the gene was highlighted in the sub-network of stress-treated tissues.

### Functional analysis tools

The transcription factor family and kinase family classifications were predicted by the standalone program iTAK (http://bioinfo.bti.cornell.edu/cgi-bin/itak/index.cgi) based on the rules from the PlnTFDB^40^ and PlantsP databases^41^. A total of 3305 transcription factors and 1598 kinases were identified. GO was based on customized enrichment analysis in agriGO^42^.

### Cis-element significance analysis

The cis-element significance test is a statistical algorithm based on Z score and P-value filtering^43^,^44^, and motifs with a P-value less than 0.05 were significantly enriched in a regulatory module^43^,^45^. Here, cis-elements were scanned in the 3 kb promoter region of cotton genes, and the Z score was calculated according to the equation


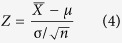



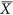
, sum value of a motif in the promoters of a list of genes. μ, mean value of the same motif in 1000 random lists of genes with same scale. σ, standard deviation of the 1000 mean value based on random selection.

### Custom GO analysis

The GO annotation of CottonGen was the BGI-CGP version. We expanded the GO annotation range using several tools. The blast2GO software predicted the GO annotation of *G. arboreum* proteins by InterProScan. The Pfam search tool predicted protein function domains, and the GO IDs were translated by Pfam domain IDs. The GO annotations were also predicted through *Arabidopsis* orthologue GO annotation. Finally, 72,812 GO-gene annotation entries covered 22,938 genes in *G. arboreum*.

We uploaded the integrated GO annotation version in agriGO and used the custom GSEA tool for the GO enrichment analysis of the 1752 DEGs by the Fisher test and Yekutieli multi-test adjustment method. The GO entries with a q-value (FDR) less than 0.05 were considered to be significantly enriched.

### Phylogenetic tree of the JAZ family

The bidirectional blast alignment between the protein sequences of 13 *Arabidopsis* JAZ family members and all proteins of *G. arboreum* was analysed. The best result was chosen, and 14 genes, including 7 different JAZ members, were found in *G. arboreum.* Then, 14 protein sequences of *G. arboreum* and 13 protein sequences of *Arabidopsis* were collected for multiple sequence alignment and phylogenetic analysis through MEGA (v5.02). Here, clustalw analysis was performed with the default parameters, and the neighbour-joining method was used to construct the phylogenetic tree. In parameter selection, there were 1000 bootstrap replicates, the substitution model was the Poisson model, Rates among Sites was Uniform rates, and Pattern among Lineages was Same (Homogeneous).

### Orthologue identification in *Arabidopsis*

Bidirectional blast alignments were conducted for the analysis of protein sequences in *G. arboreum* and *Arabidopsis*. Our criteria for the ortholog search were as follows: the top three hits in each bidirectional blast alignment were selected as the best orthologous pairs; in addition, pairs with an e-value less than 1E-55 were regarded as secondary orthologous pairs^46^. Supplementary Table 2 lists the results of the ortholog search, including for MYB46, VND6, KNAT7, and REV.

### Search and visualization platform

The network search function is based on MySQL, Apache and PHP scripts. Cytoscape.js, an open source javascript package, can dynamically display the components, construction and variation of the network.

## Additional Information

**How to cite this article**: You, Q. *et al*. Co-expression network analyses identify functional modules associated with development and stress response in *Gossypium arboreum. Sci. Rep.* **6**, 38436; doi: 10.1038/srep38436 (2016).

**Publisher's note:** Springer Nature remains neutral with regard to jurisdictional claims in published maps and institutional affiliations.

## Supplementary Material

Supplemental Materials

Supplementary Dataset S1

Supplementary Dataset S2

Supplementary Dataset S3

## Figures and Tables

**Table 1:** Details of RNA-seq sample resources.

Name	Tissue	Sample information	Source	Reference
Fibre_10 dpa	Fibre	10 dpa	SRX062247	NCBI (2011)
Fibre_20 dpa		20 dpa	SRX062251	
Seed_10 dpa	Seed	cotton seeds: 10 dpa	SRR617074, SRR617075, SRR617076	Genome Biol Evol. 2014 Mar;6(3):559-71 (PMID: 24558256)
Seed_20 dpa		cotton seeds: 20 dpa	SRR617071, SRR617072, SRR617073	
Seed_30 dpa		cotton seeds: 30 dpa	SRR617068, SRR617069, SRR617070	
Seed_40 dpa		cotton seeds: 40 dpa	SRR617065, SRR617066, SRR617067	
Seeds_germinated_5 h	Seedling	germination stages: 5 h	SRX323746	PLoS One. 2013 Sep 20;8(9):e75323 (PMID:24073262)
Seeds_germinated_15 h		germination stages: 15 h	SRX323748	
Seeds_germinated_30 h		germination stages: 30 h	SRX323750	
Leaf_3 cm	Leaf	seventh fully expanded true leaf (3 cm in length)	SRX170955, SRX172454, SRX172473	Heredity (Edinb). 2013 Feb;110(2):171-80 (PMID:23169565)
Root_CK	Root	plain water		PLoS One. 2013;8(1):e54762 (PMID:23382961)
Root_PEG		3 h with 17% PEG 6000		
Root_NaCl		3 h with 150 mMNaCl		
Stem_CK	Stem	plain water		
Stem_PEG		3 h with 17% PEG 6000		
Stem_NaCl		3 h with 150 mMNaCl		
Leaf_CK	Leaf	plain water		
Leaf_PEG		3 h with 17% PEG 6000		
Leaf_NaCl		3 h with 150 mMNaCl		
