# Supplementary material for: Co-expression network analyses identify functional modules associated with development and stress response in Gossypium arboreum
Source: Sci Rep. 2016 Dec 6;6:38436. doi: 10.1038/srep38436 (PMC5138846; doi:10.1038/srep38436)
Supplement: Supplemental Materials [file srep38436-s1.pdf]

Title of Article:

Co-expression network analyses identify functional modules associated with development and stress response in *Gossypium arboreum*

Qi You[1], Liwei Zhang[1], Xin Yi[1], Kang Zhang[1], Dongxia Yao[1], Xueyan Zhang[2], Qianhua Wang[2], Xinhua Zhao[2], Yi Ling[1], Wenying Xu*[1], Fuguang Li*[2], Zhen Su*[1]

[1]State Key Laboratory of Plant Physiology and Biochemistry, College of Biological Sciences, China Agricultural University, Beijing 100193, China

[2]State Key Laboratory of Cotton Biology, Institute of Cotton Research, Chinese Academy of Agriculture Sciences (CAAS), Anyang, Henan 455000, China

* Corresponding author

Zhen Su

e-mail: zhensu@cau.edu.cn; fax: +86-10-62731380

Fuguang Li

e-mail: aylifug@hotmail.com; fax: +86-372-2562256

Wenying Xu

e-mail: x_wenying@yahoo.com; fax: +86-10-62731380

Supplemental materials and methods

1. methods

I. Hierarchical Clustering of gene and tissues

II. Experiment materials

III. ROC curves of co-expression networks

2. Tables

Supplementary Table 1. Data information, quality and mapping ratios of RNA-seq samples

Supplementary Table 2. Orthologue search criteria and annotation

Supplementary Table 3. Primer list for real-time RT-PCR

3. Figures and legends

Figure S1. Co-expression network construction strategy and conditional multi-dimension analysis

Figure S2. Phylogenetic tree of the JAZ family in *Arabidopsis* and *G. arboreum*

Figure S3. Q-RT-PCR validation for selected JAZ genes

Figure S4. Proper selection for function module size

Figure S5. Gene expression profiling comparison between *G. arboreum* and *G. hirsutum*

Figure S6. ROC curves of co-expression networks with different PCC and MR thresholds

Figure S7. Comparisons between global, tissue-specific and stress-treatment co-expression network

Figure S8. Statistical result of nodes and edges in global, tissue-specific and stress-treatment co-expression network

4. Supplementary datasets

Dataset S1. 1752DEGs between vegetative and reproductive stages

Dataset S2. Salt stress response module

Dataset S3. CPM functional modules

5. References

I. Hierarchical clustering of genes and tissues

An R script was used for cluster analysis. The command line reference is the "R & Bioconductor Manual" (http://manuals.bioinformatics.ucr.edu/home/R_BioCondManual).

(1) Classification of the samples into vegetative and reproductive groups (Figure 2A)

The Spearman correlation method was applied to calculate the pairwise correlation coefficient of the 29 samples. The minimum coefficient value is 0.7, and the significance test p-value is less than 2.2e-16. Then, the expression profiles of the 29 tissues were clustered by the complete linkage method. Finally, the hierarchical dendrogram (vertical) was reordered by the tissue cluster result (the horizontal dendrogram is a replicate result of the vertical dendrogram). According to the hierarchical clustering, the 29 samples were classified into two main groups. The gene expression profiles of the 12 seed samples (from 10 dpa to 40 dpa) were closer to the fibre sample profiles, while the root, stem, leaf and seedling samples were more similar to one another. This method was described in a previous paper on integrated network analysis in grapevine[1].

Spearman correlation coefficient:

$$\rho = 1 - \frac{6\sum d_i^2}{n(n^2-1)} \quad , \quad d_i = x_i - y_i \quad \text{Equation 1}$$

X and Y represent the FPKM values of a gene in two different tissues, and n stands for the number of genes in *Gossypium arboreum* (here, n = 41331).

Correlation significance test:

$$t = \frac{r_{xy}\sqrt{\mathrm{n}-2}}{\sqrt{1-r_{xy}^2}} \quad \text{Equation 2}$$

Here r represents the Spearman correlation coefficient, and n stands for the number of genes in *G. arboreum* (here, n = 41331).

(2) The 1752 DEGs between vegetative and reproductive organs (Figure 2B)

First, TTEST was calculated between the gene expression profiles of vegetative and reproductive groups (Tails = "one-tailed distribution", Type = "Two-sample unequal

variance (heteroscedastic)"). Then, the FDR of each gene's p-value was adjusted by the Benjamini-Hochberg (HB) method, and the FPKM fold change (FC) of each gene was compared to obtain the average expression values of the vegetative and reproductive organs. Finally, 1752 genes with q-values less than 0.001 and $|log2(FC)| > 1.7$ were considered differentially expressed.

All gene expression values among the 29 RNA-seq samples were centred (mean) and scaled before hierarchical clustering. The Pearson correlation method was applied to calculate the pairwise correlation coefficients of the 1752 gene expression profiles. The minimum coefficient value was 0.49, and the significance test p-value was less than 1e-7. The Spearman correlation method was applied to calculate the pairwise correlation coefficients of the 29 samples. The minimum coefficient value was 0.52, and the significance test p-value as less than 1.9e-3. Then, the 1752 genes and 29 tissues were clustered by the average linkage method.

(3) The 162 transcription regulators in the 1752 DEGs (Figure 3A)

The hierarchical clustering of the 162 TFs was performed similarly to the method for the 1752 DEGs described above. The cutoff of the gene correlation coefficient (Pearson) was 0.46, and the significance test p-value was less than 1e-7. The cutoff of the tissue correlation coefficient (Spearman) was 0.55, and the significance test p-value was less than 9.97e-4.

(4) Cluster analysis of salt stress response genes (Figure 5A)

The 5129 genes with $|log2(FC)| \geq 1$ were regarded as up-regulated in the root after salinity treatment. The numeric matrix (root_up) of the gene expression values contains 9 columns (water stress and control samples in root, stem and leaf) and 5129 rows (up-regulated genes in root). The hierarchical clustering method was similar to that used for the 1752 DEGs described above. The cutoff of the gene correlation coefficient (Pearson) was 0.2, and significance test p-value was less than 1e-7. The cutoff of the gene correlation coefficient was 0.27 (Spearman), and the significance test p-value was less than 0.24.

(5) Cluster analysis of *GhKNL1* and orthologous co-expressed genes (Figure S5)

The tool Cluster3.0 performed hierarchical clustering of 52 co-expressed genes in the sub-network of Cotton_A_28415 (Figure S5A) and 44 co-expressed genes in the sub-networks of Gh_D08G1910 (*GhKNL1*) and Gh_A08G1599, respectively (Figure S5B). The correlation coefficient was calculated by the Pearson method. The cutoff of the gene correlation coefficient was 0.63, and the significance test p-value was less than 5e-7 in the Cotton_A_28415 co-expressed gene clustering treeview. The cutoff of the gene correlation coefficient was 0.27, and the significance test p-value was less than 0.038 in the *GhKNL1* co-expressed gene clustering treeview.

II. Experiment materials

Plant material and growth conditions

Cotton (*G. arboreum* L. cv. Shixiya) seeds were immersed in hot water (80°C) for 2h, stayed in room temperature for 2 days, and then placed for germination on sterilized soil in plates maintained under the following conditions: 28/25°C, 12/12 h of light/darkness, and relative humidity of 80%. 3-day-old germinated plants were transferred to black plastic tanks filled with nutrient solution[2] and kept growing until they had produced 6-7 leaves. The cotton seedlings were treated with 150 mM NaCl and 17% PEG 6000, respectively, and water as mock. After exposing the seedlings to different solutions for 3h, the leaf, stem (including hypocotyl), and root tissues were harvested at the same time.

Arabidopsis thaliana (Col-0 and transgenic lines) seeds were surface sterilized with 2% sodium hypochlorite, washed in sterile water five times, sown on MS-agar Petri plates, and placed in the dark at 4 °C for 3 days. Seedlings were incubated in a growth chamber (16-h light /8-h dark at 22 °C). Plants were continued on MS medium or transferred to soil, depending on the requirement for the experiments.

RNA isolation and Q-RT-PCR analysis

All the 9 cotton tissue samples were homogenized in liquid nitrogen before isolation of RNA. Total RNA was isolated using a modified CTAB method and purified using Qiagen RNeasy columns (Qiagen, Hilden, Germany).

Reverse transcription was performed using an M-MLV kit (Invitrogen). The samples, 10 µl each containing 2 µg of total RNA and 20 pmol of random hexamers (Invitrogen), were maintained at 70°C for 10 min to denature the RNA and then chilled on ice for 2 min. The reaction buffer and M-MLV enzyme (20 µl of the mixture contained 500 µM dNTPs, 50 mM Tris-HCl (pH 8.3), 75 mM KCl, 3 mM MgCl2, 5 mM dithiothreitol, 200 units of M-MLV, and 20 pmol random hexamers) was added to the chilled samples and the samples maintained at 37°C for 1 h. The cDNA samples were diluted to 8 ng/µl for RT-PCR analysis.

For Q-RT-PCR, assays were performed in triplicate on 1 µl of each cDNA dilution using the SYBR Green Master Mix (PN 4309155, Applied Biosystems) with an ABI 7500 sequence detection system as prescribed in the manufacturer's protocol (Applied Biosystems). The gene-specific primers were designed using PRIMER3 (http://frodo.wi.mit.edu/primer3/input.htm). The amplification of 18S rRNA was used as an internal control to normalize all data (forward primer, 5'-CGGCTACCACATCCAAGGAA-3'; reverse primer, 5'-TGTCACTACCTCCCCGTGTCA-3'). The gene-specific primers are listed in Supplementary Table 3. The relative quantification method (ΔΔCT) was used for quantitative evaluation of the variation between replicates.

RT-PCR for transgenic *Arabidopsis* lines

To detect the expression level of *GaJAZ1a* in transgenic *Arabidopsis* lines, RT-PCR method was performed. Total RNA extracted from transgenic *Arabidopsis* plants was denatured at 70 °C for 5 min and reverse transcribed at 42 °C for 60 min using AMV reverse transcriptase (Promega, Madison, WI, USA). PCR amplification was performed using the *GaJAZ1a* primers (P1, 5'-ATGTTTGGTTCACCGGAATATACAT-3'; P2, 5'-CTATCCCTTTCTCTTCTCG-3') corresponding to a 651bp fragment. The amplification program consisted of 5 min at 94 °C for initial denaturation, 30 cycles for 1 min at 94 °C, 1 min at 55 °C, 1 min at 72 °C, and 10 min at 72 °C for extension.

Construction of transgenic *Arabidopsis* lines

The *GaJAZ1-like1* gene was cloned into the binary vector super-1300 controlled by the CaMV 35S promoter. The recombinant plasmids were then introduced into *Agrobacterium tumefaciens* EHA105 strain following the freeze–thaw method. Transgenic *Arabidopsis* plants were obtained by the floral dipping method [3]. The concentration of the selected antibiotic, hygromycin B, was 25 mg/L.

Salt treatment assays in transgenic *Arabidopsis* plants

Salt treatments were performed on seedling stages on plates or in soil. Seeds of 35S:: *GaJAZ1-like1* transgenic lines and WT plants were germinated on MS medium. Five days after germination, seedlings from each line were carefully transferred to new MS media with 150mM NaCl for treatment.

Water was withheld for 4 weeks and plants were then well irrigated with NaCl solution (350mM) applied at the bottom of the pots. When the soil was completely saturated with salt solution, free NaCl solution was removed and the plants were cultured under normal conditions.

Proline measurements

Free proline contents of transgenic *Arabidopsis* plants were measured. Fresh leaf tissue (0.5 g) was extracted in 5 mL of 3% sulphosalicylic acid at 95 °C for 15 min. After filtration, 2 mL of supernatant was transferred to a new tube containing 2 mL of acetic acid and 2 mL of acidified ninhydrin reagent. After 30 min of incubation at 95 °C, 5 mL of toluene was added to the tube with full shaking to extract red products. The absorbance of the toluene layer was determined at 532 nm.

Chlorophyll content measurement

Leaf chlorophyll were extracted in dimethylsulphoxide (DMSO) and measured by absorbance at 663 nm and 645 nm using a spectrophotometer (SmartSpec™ 3000, Bio-Rad, USA) [4]. Transgenic and WT *Arabidopsis* plants were treated with 350mM NaCl and the measurements were performed 24h before and after the treatment.

III. ROC curves of co-expression networks

To further increase the credibility of the co-expression network, we set strict parameters to filter out poor co-expression gene pairs. We extracted GO terms to assess co-expression networks with different cut-off values of PCC and MR. As the terms associated with too many genes have less informative annotations, thus we used the 240 BP terms of GO associated with >4 and <20 genes to assess the networks [5]. Here we showed the test results of different co-expression networks in *G. arboreum*. The highest 5% PCC value of all positive co-expression gene pairs was 0.65, we selected co-expression networks with thresholds of PCC>0.65, PCC>0.75 and PCC>0.85 to predict gene function (GO terms) and generated receiver operating characteristic (ROC) curves. As a result, the AUC of co-expression network with 0.65 cut-off is better than the other two (Figure S6B). In addition, co-expression networks with thresholds of MR top3+MR ≤ 30, MR top3+MR ≤ 50 and MR top3+MR ≤ 100 were tested and the network with MR top3+MR ≤ 30 was better (Figure S6A). ROC corves of tissue-specific network (Figure S6C) and stress-treatment network (Figure S6D) showed the similar tendency. The ROC curves and AUC values were calculated by pROC package in R script.

Supplementary Table 1 Data information, quality and mapping ratios of RNA-seq samples

Source	Total reads	Map reads	Final map ratio
SRX062247 (single end)	11163357	9023994	80.8%
SRX062251 (single end)	10384316	8339296	80.3%
SRR617074 (single end)	16988383	12452193	77.7%
SRR617075 (single end)	24924413	15979479	71.6%
SRR617076 (single end)	19535838	11699437	68.4%
SRR617071 (single end)	18788643	12397333	72.4%
SRR617072 (single end)	19421293	11597587	71.1%
SRR617073 (single end)	26163876	18080546	74.0%
SRR617068 (single end)	29554283	19470442	73.1%
SRR617069 (single end)	17955647	10794969	68.5%
SRR617070 (single end)	14533731	9302757	69.3%
SRR617065 (single end)	22778582	15897320	74.5%
SRR617066 (single end)	17586367	11171103	71.1%
SRR617067 (single end)	26124332	15915347	72.4%
SRX323746 (single end)	30426341	27628069	90.8%
SRX323748 (single end)	65824214	59474473	90.4%
SRX323750 (single end)	30080344	27136293	90.2%
SRX170955 (single end)	9025496	7589866	84.1%
SRX172454 (single end)	5444466	4821902	88.6%
SRX172473 (single end)	6988156	6273066	89.8%
Root_CK (paired end)	55986464	46498061	83.1%
Root_PEG (paired end)	54455082	48420621	88.9%
Root_NaCl (paired end)	61212534	55810480	91.2%
Stem_CK (paired end)	26755556	23857640	89.2%
Stem_PEG (paired end)	38519182	34635525	89.9%
Stem_NaCl (paired end)	29599346	26740994	90.3%
Leaf_CK (paired end)	26711112	23901463	89.5%
Leaf_PEG (paired end)	27555556	23602773	85.7%
Leaf_NaCl (paired end)	26733332	22773223	85.2%

Supplementary Table 2 Orthologue search criteria and annotation

G. arboreum	*Arabidopsis* orthologue	Blast e-value	Annotation
Cotton_A_12989	AT5G12870	1E-49	MYB46
Cotton_A_23892	AT5G62380	2E-67	VND6
Cotton_A_28415	AT1G62290	2.2E-120	KNAT
Cotton_A_07061	AT5G60690	0	REVOLUTA
Cotton_A_00715	AT4G34610	1E-121	BLH6
Cotton_A_07124	AT1G12260	1.5E-122	VND4
Cotton_A_40148	AT4G18960	1.2E-94	AGAMOUS
Cotton_A_07375	AT4G13640	1.3E-98	G2-like TF
Cotton_A_11862	AT1G19180	1e-39	JAZ1

Supplementary Table 3 Primer list for real-time RT-PCR

Unigene ID	Forward	Reverse
Cotton_A_11862 (*GaJAZ1a*)	TACCCATTGCTCGAAGAGCT	GCCGTTTATTGGGTATGGTG
Cotton_A_27840 (*GaJAZ1b*)	CCTACCTCAAAACCGGTTCA	AACCGATGCAGTGAAGCTCT
Cotton_A_36075 (*GaJAZ3a*)	CGTATGCAAGACCACAGGAA	TCCCATGGTATTTGCCAATT
Cotton_A_18896 (*GaJAZ5b*)	GGAGATCATGGCCGTAGCTA	TTTTTCCATGGAAGAGTCGG

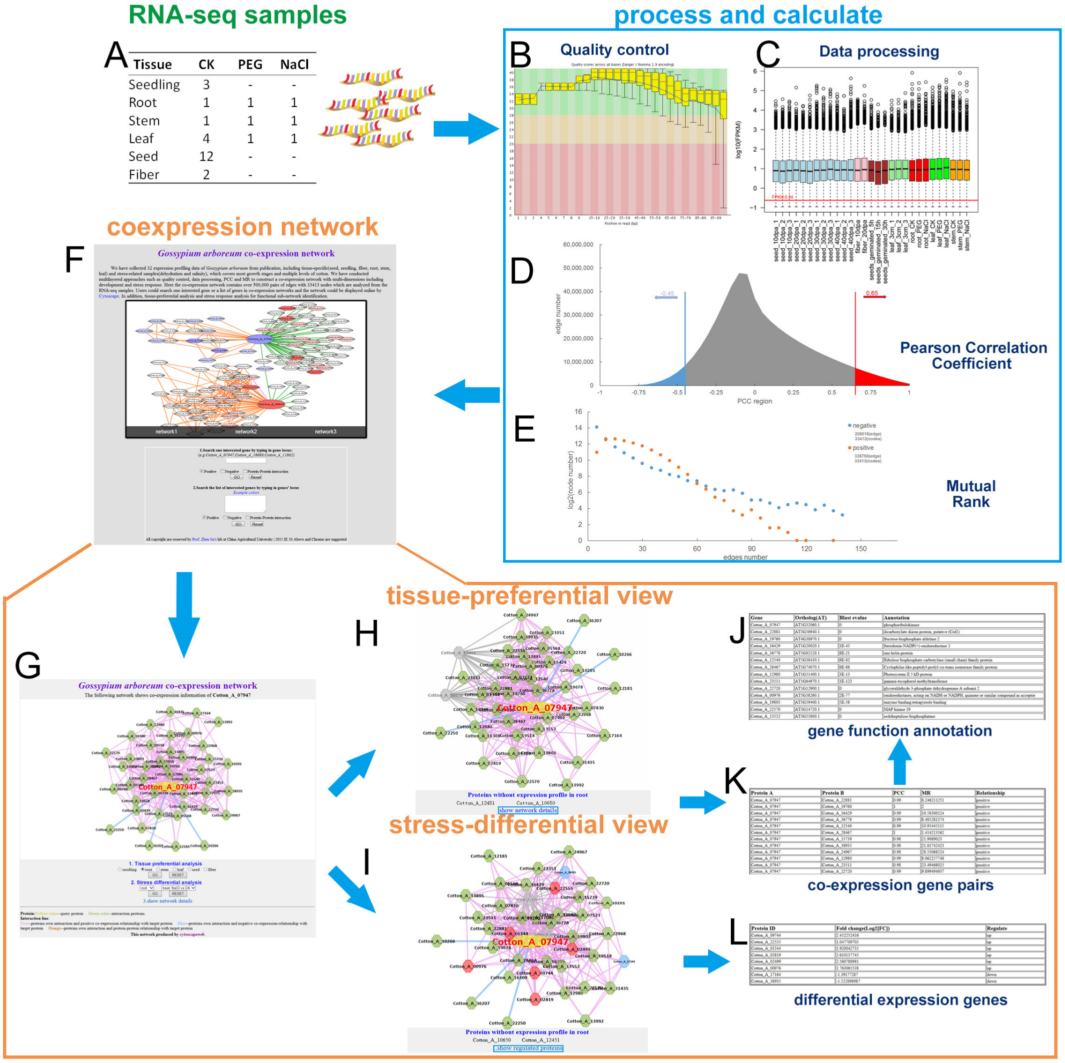

Figure S1. Co-expression network construction strategy and conditional multi-dimension analysis

(A) The table lists 29 RNA-seq samples for co-expression network construction. **(B)** An example of read quality evaluated by FastQC tool. The x-axis represents the length of reads in an RNA-seq sample, and the y-axis represents the sequencing quality of the nucleotide. If the bottom number associated with every yellow box is greater than 20, the quality of the RNA-seq sample is acceptable. **(C)** The boxplot displays the distribution of all genes' FPKM values among the 29 different samples. The x-axis lists the 29 RNA-seq samples, and the y-axis is the gene expression value ($LOG_{10}FPKM$). Here, the red horizontal line represents the threshold for the minimum FPKM of the expressed gene, at 0.24 FPKM. **(D)** The PCC distribution of all gene pairs in *G. arboreum*. The lowest 5% and highest 5% PCC values, namely -

0.45 and 0.65, were set as thresholds, and gene pairs with PCC values in the relevant region were regarded as co-expressed. **(E)** The scatter plot contains the statistical results for the nodes and edges of the MR co-expression network. The y-axis represents nodes and the x-axis edges in the MR network. An orange dot indicates that there are y nodes with x edges in the positive co-expression network (PCC > 0). A blue dot indicates that there are y nodes with x edges in the negative co-expression network (PCC < 0). **(F)** A platform for network search and visualization. Users can search a single positive or negative co-expression network of a gene and can simultaneously search the positive and negative co-expression network. A list of genes is provided for network analysis. **(G)** The network is displayed by the Cytoscape web tool. The yellow node with red text is the query gene (Cotton_A_07947), and a green node represents the co-expressed gene. A pink or blue line connects two genes with positive or negative co-expression relationships, respectively. Then, "Tissue preferential analysis" and "Stress differential analysis" are linked to the gene expression view analyses. **(H)** The gene tissue-preferential expression view provides six growth stages, and users can select one of them to overlay gene expression. Grey- and green-coloured nodes represent un-expressed and expressed genes, respectively, in the tissues. The un-expressed genes are listed download. "Show network details" is linked to a secondary web page showing co-expression gene pair annotation. **(I)** The gene stress-differential expression view shows differential expression in the root, stem and leaf after PEG or salt treatment. Grey nodes represent un-expressed genes in the tissues; a red node indicates up-regulated gene expression after the stress treatment; a blue node indicates down-regulated gene expression after the stress treatment; a green node indicates a gene without significant differences in expression level. The un-expressed genes will be listed in a download. "Show regulated proteins" links to a DEG list. **(J)** A web page for co-expressed gene annotation, including orthologous gene ID, BLAST search score and TAIR10 annotation *Arabidopsis*. **(K)** A web page for details of the co-expressed gene pairs in a sub-network. The table lists gene ID, PCC score, MR value and co-expression relationship. **(L)** A web page for DEGs. The table lists the fold

changes of gene expression values after stress treatment.

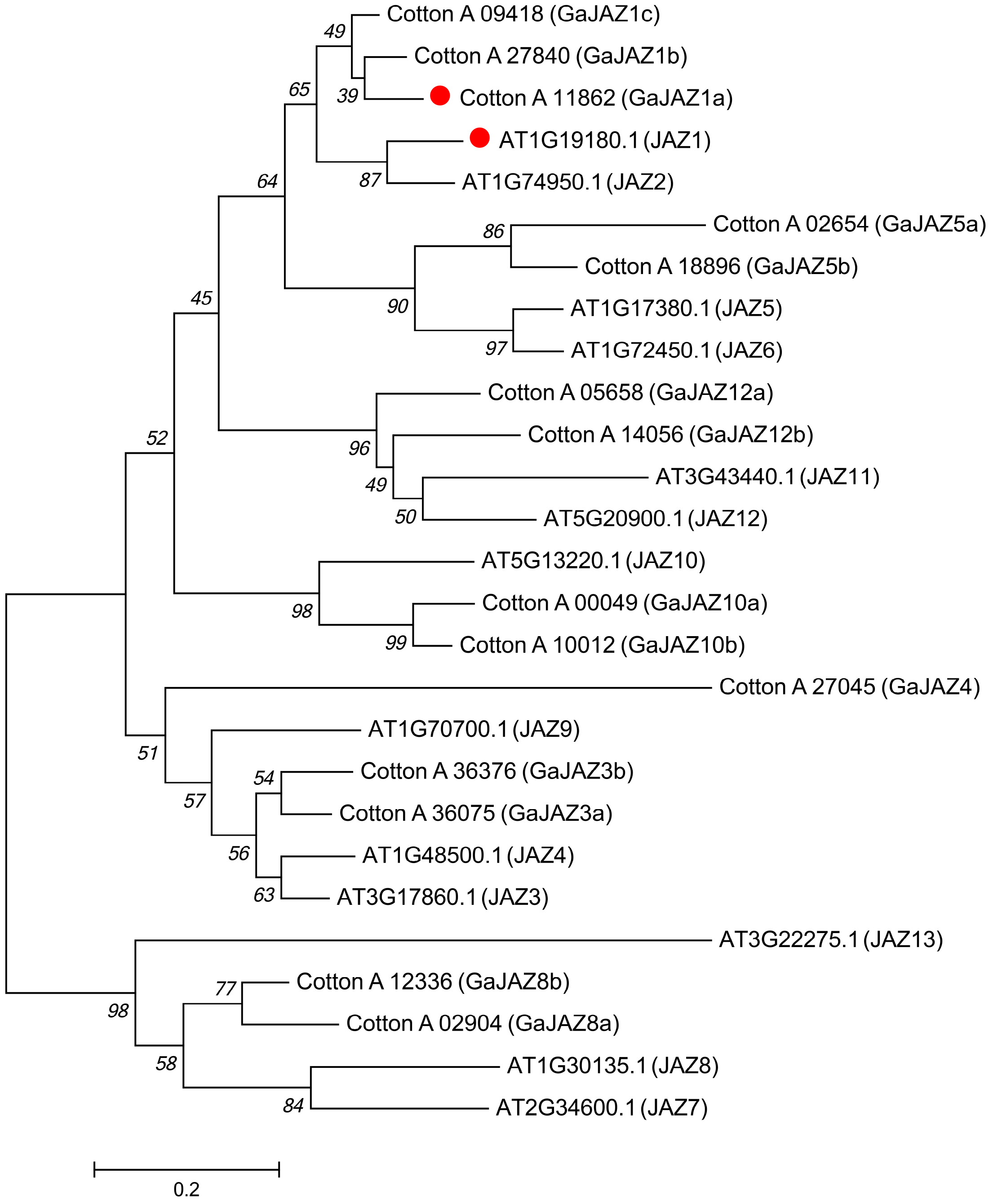

Figure S2. Phylogenetic tree of the JAZ family in *Arabidopsis* and *G. arboreum*

The JAZ family phylogenetic tree contains 14 cotton genes (such as *GaJAZ1a*) and 13 *Arabidopsis* genes (such as *JAZ1*). In contrast to the 13 JAZ members in *Arabidopsis*, there are seven JAZ family members in *G. arboreum*, including *GaJAZ1, GaJAZ3, GaJAZ4, GaJAZ5, GaJAZ8, GaJAZ10* and *GaJAZ12*. Several JAZ members have multiple copies (locus gene ID), which we manually distinguish by adding "a, b, and c", such as *GaJAZ1a, GaJAZ1b* and *GaJAZ1c*.

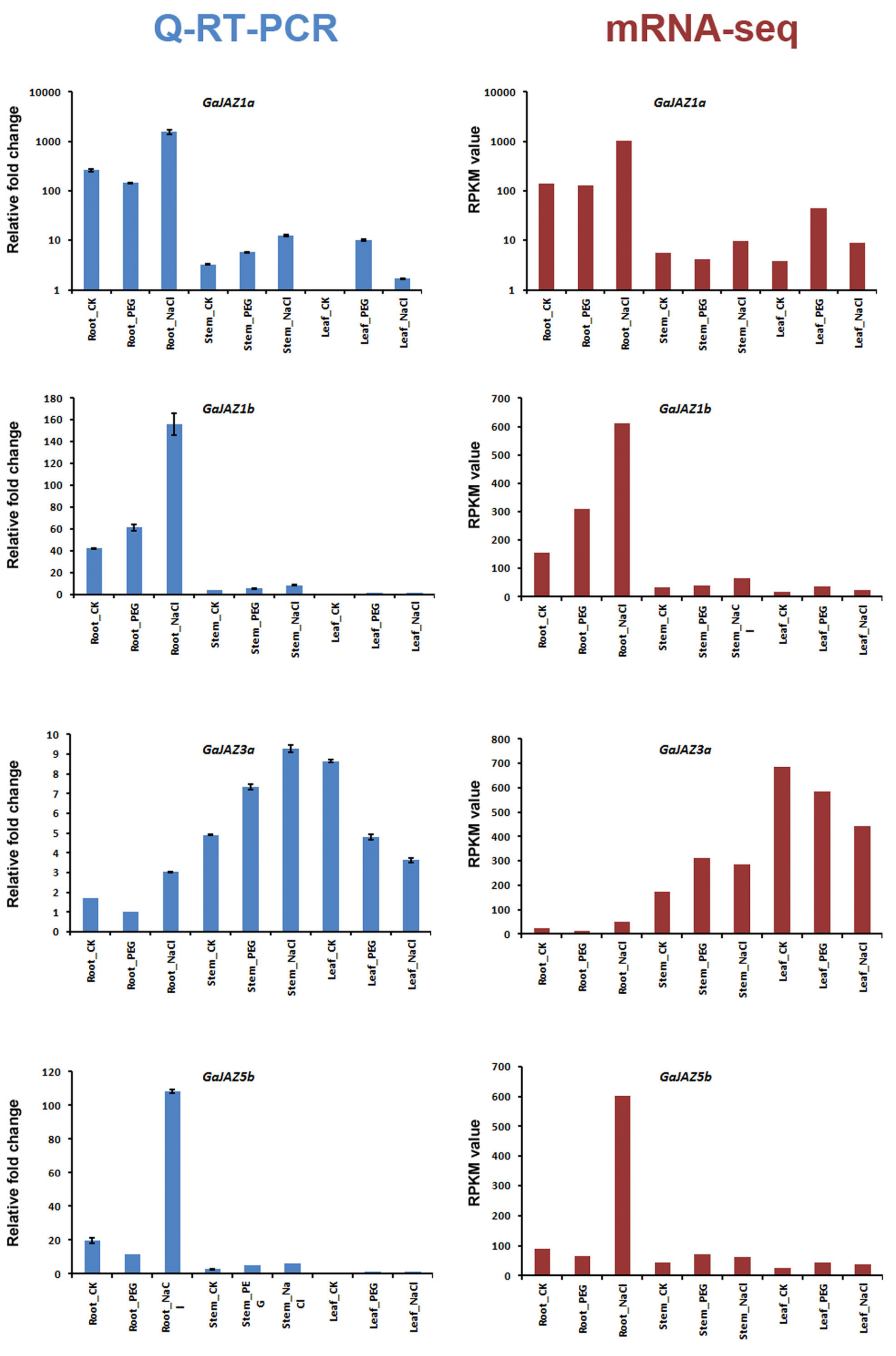

Figure S3. Q-RT-PCR validation for selected JAZ genes

Four *GaJAZ* genes were selected for Q-RT-PCR to validate the expression patterns in different samples and treatments. The blue bars on the left side represent the relative intensity of Q-RT-PCR from independent biological replicates, and the red bars on the right side represent the expression levels (RPKM) of the transcripts.

The *GaJAZ* genes are: Cotton_A_11862 -- *GaJAZ1a*; Cotton_A_27840 -- *GaJAZ1b*;

Cotton_A_36075 -- *GaJAZ3a*; Cotton_A_18896 -- *GaJAZ5b*. The Q-RT-PCR primers for each transcript are listed in Table S3.

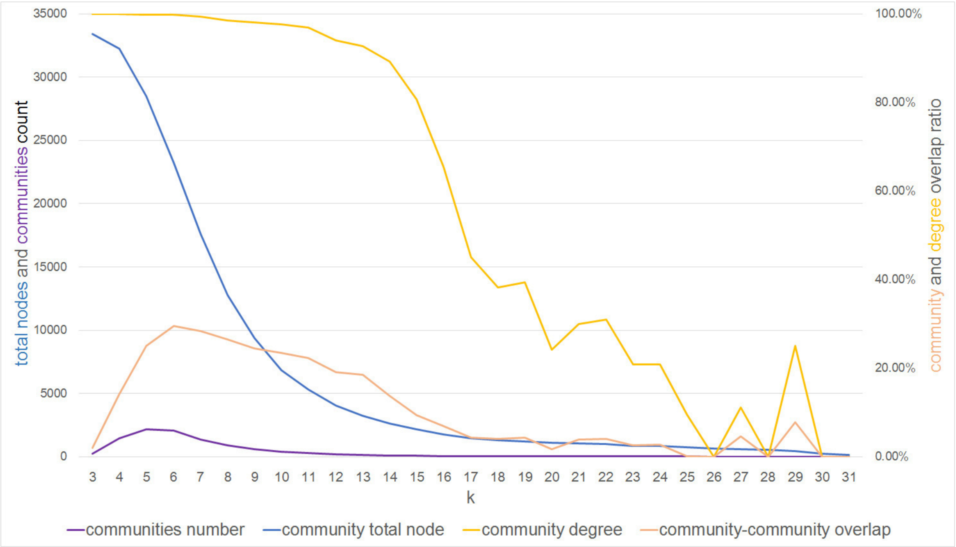

Figure S4. Proper selection for function module size

The tool CFinder was applied to calculate communities of different k-clique sizes (from k = 3 to k = 31). Statistics of the communities of different k-clique sizes, including community number, community degree, community-community overlap and community total node were compared. Here, community number represents the number of communities of a selected k-clique size; community degree represents the number of other communities overlapping with a selected community; community-community overlap represents the number of nodes contained by two overlapping communities; community total nodes represents the total genes contained in a given k-clique size. The left y-axis represents the community number and community total nodes, while the right y-axis represents the community degree ratio (number of communities with overlapped communities/number of total communities) and community-community overlap ratio (number of nodes contained by two overlapping communities/community total node). The clique size k = 6 contains the most communities, more than 56% of *G. arboreum* coding genes, the highest community-community overlap ratio and the top community degree ratio. We selected the k = 6 clique because it offers more possible functional modules (communities), more gene coverage and more community overlap (mimicking crosslinks of biological processes).

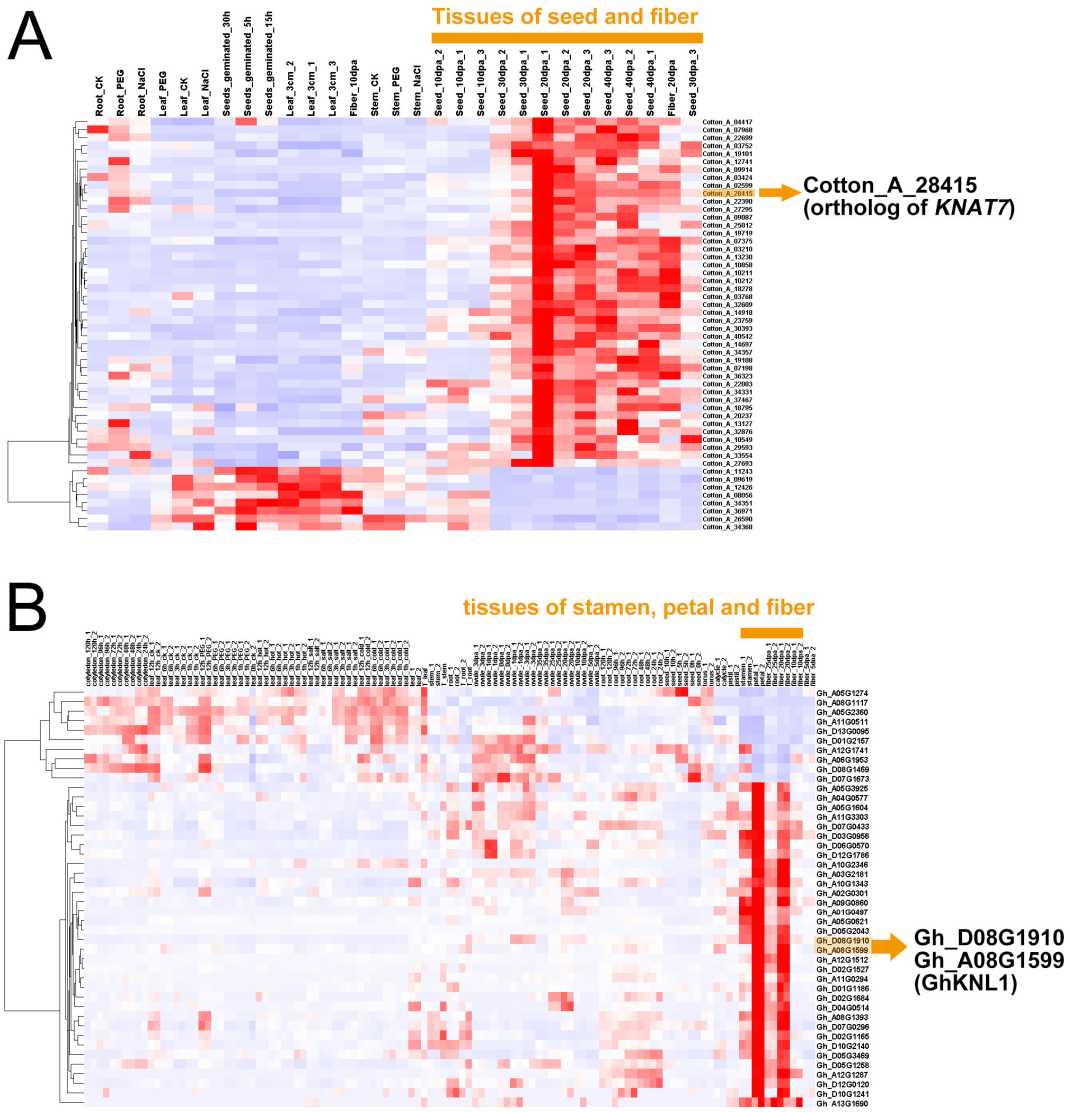

Figure S5. Gene expression profiling comparison between *G. arboreum* and *G. hirsutum*

(A) The expression profiling heatmap of the MR network members of Cotton_A_28415 was generated by the hierarchical clustering method. Genes are clustered vertically, and tissues are clustered horizontally. A red box represents a gene highly expressed in a sample; a white box represents a gene without significant expression changes; a blue box represents a gene lowly expressed in a sample. (B) The 115 public RNA-seq samples of *G. hirsutum*, including different tissues (root, stem, leaf, cotyledon, calycle, pistil, stamen, petal, torus, ovule, fibre and seed) and stress-treated leaf samples (dehydration, salinity, heat and cold) were collected from NCBI. The top 44 genes shared high co-expression relationships (positive or negative) with Gh_D08G1910 and

Gh_A08G1599. The details of the PCC value calculation have been published. All of these co-expressed genes are used for hierarchical cluster analysis, and the heatmap displays the results. The meanings of the coloured boxes are the same as in Figure S5A. The criteria of the hierarchical clustering method are similar to the criteria used for the 1752 DEGs.

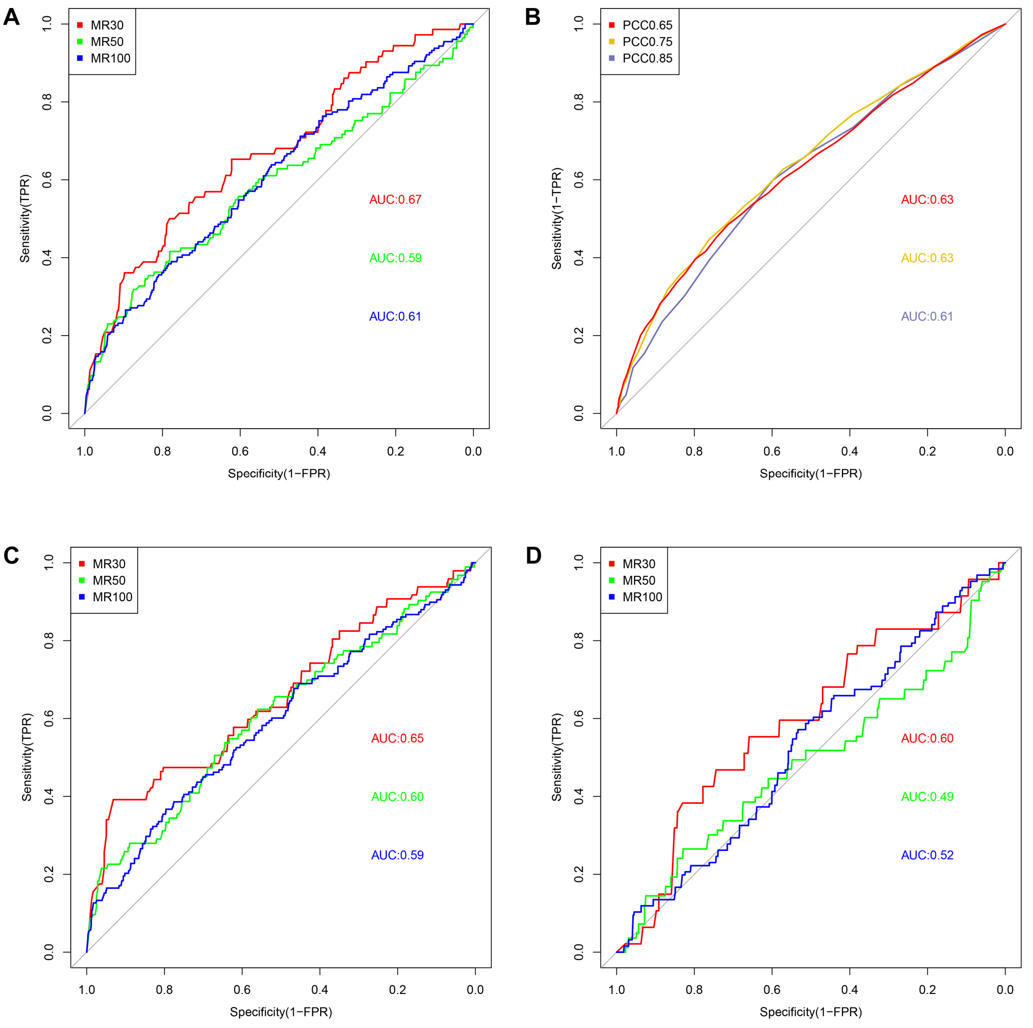

Figure S6. ROC curves of co-expression networks with different PCC and MR thresholds

(A) A plot of true-positive rate [TP/(TP + FN)] against false-positive rate [TN/(FP + TN)] of global possible co-expression networks with different MR thresholds (MR top3+MR ⩽ 30, MR top3+MR ⩽ 50, MR top3+MR ⩽ 100), where TP, FN, TN and FP are the number of true positives, false negatives, true negatives and false positives, respectively. (B) A plot of true-positive rate [TP/(TP + FN)] against false-positive rate [TN/(FP + TN)] of global co-expression networks with different PCC thresholds (PCC>0.65, PCC>0.75, PCC>0.85), where TP, FN, TN and FP are the number of true positives, false negatives, true negatives and false positives, respectively. (C) ROC curves of tissue-specific co-expression networks with different MR thresholds. (D) ROC curves of stress-treatment co-expression networks with different MR

thresholds

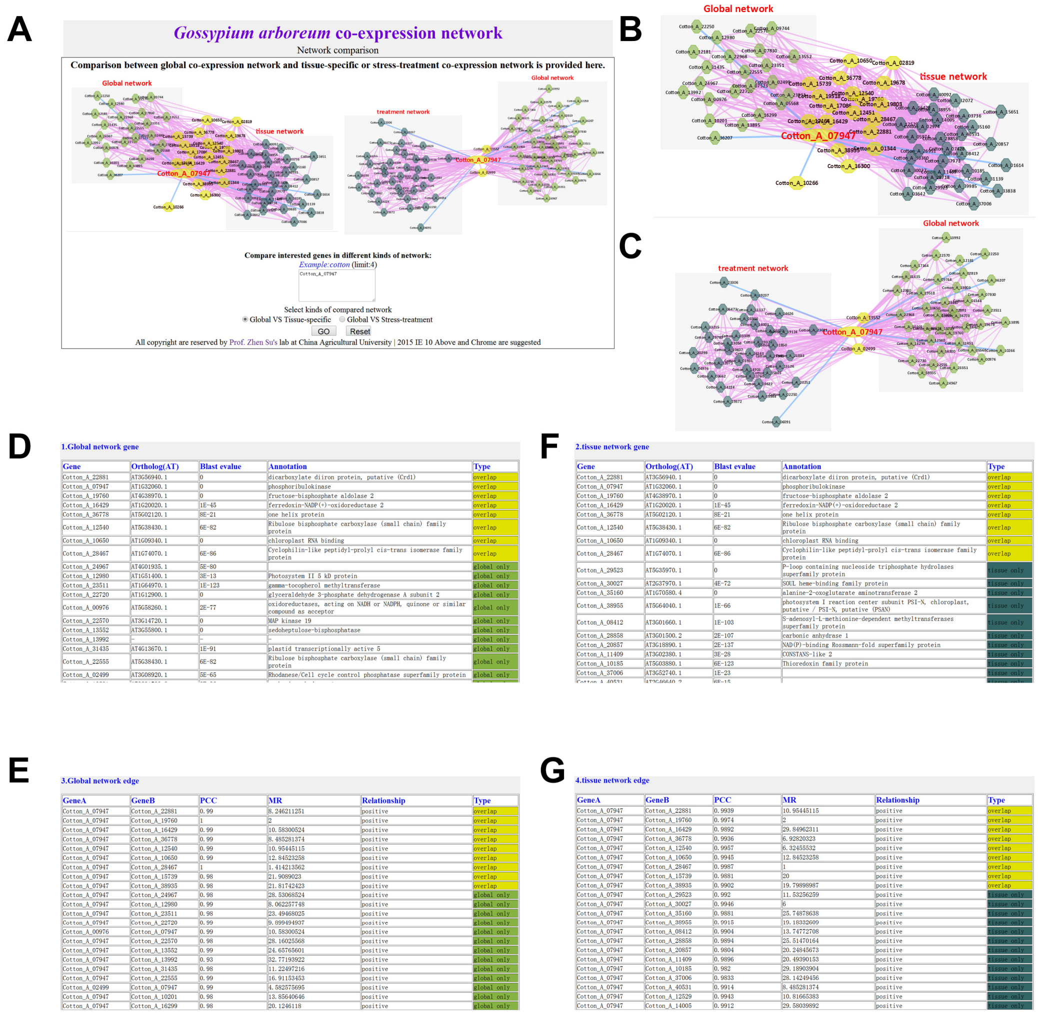

Figure S7. Comparisons between global, tissue-specific and stress-treatment co-expression network

(A) The network comparison tool. Comparison between global and tissue-specific co-expression network, and comparison between global and stress-treatment co-expression network are supported. (B) An example of comparison result between global and tissue-specific co-expression network. The nodes with yellow color stand for overlaps of the two networks, the nodes with light-green color stand for unique genes in global sub-network, and the nodes with dark-green color stand for unique genes in tissue-specific sub-network. A pink or blue line connects two genes with positive or negative co-expression relationships, respectively. (C) An example of comparison result between

global and stress-treatment co-expression network. The nodes with yellow color stand for overlaps of the two networks, the nodes with light-green color stand for unique genes in global sub-network, and the nodes with dark-green color stand for unique genes in stress-treatment sub-network. A pink or blue line connects two genes with positive or negative co-expression relationships, respectively. (D) - (E) Annotation of nodes and edges in the global network. (F) - (G) Annotation of nodes and edges in the tissue-specific network.

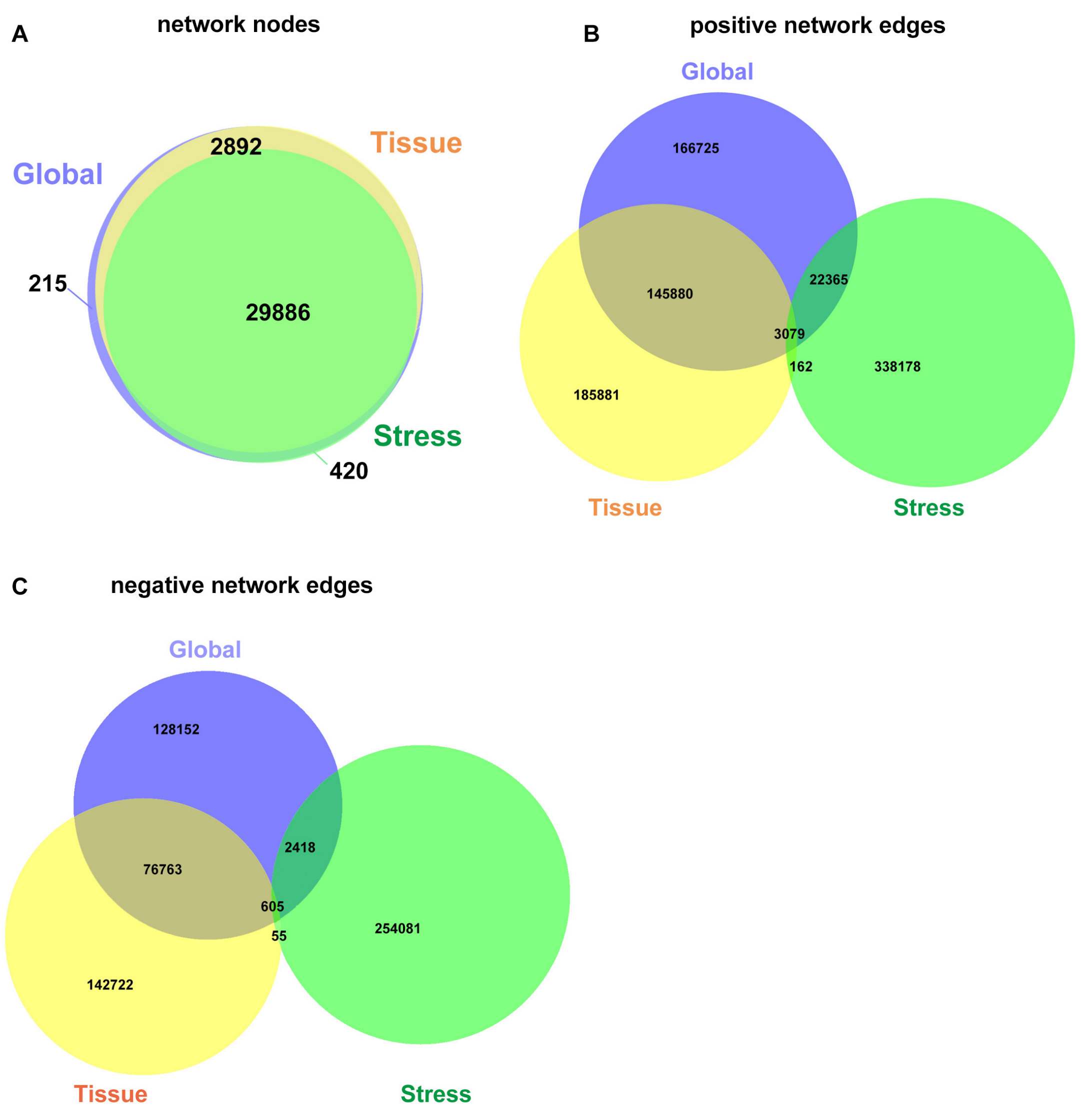

Figure S8. Statistical result of nodes and edges in global, tissue-specific and stress-treatment co-expression network

(A) Number of genes covered in the three kinds of co-expression networks. (B) Number of positive co-expressed relationships in the three co-expression networks. (C) Number of negative co-expressed relationships in the three co-expression networks.

References

1 Palumbo, M. C. *et al.* Integrated network analysis identifies fight-club nodes as a class of hubs encompassing key putative switch genes that induce major transcriptome reprogramming during grapevine development. *The Plant cell* **26**, 4617-4635, doi:10.1105/tpc.114.133710 (2014).

2 Yao, D. *et al.* Transcriptome analysis reveals salt-stress-regulated biological processes and key pathways in roots of cotton (Gossypium hirsutum L.). *Genomics* **98**, 47-55, doi:10.1016/j.ygeno.2011.04.007 (2011).

3 Clough, S. J. & Bent, A. F. Floral dip: a simplified method for Agrobacterium-mediated transformation of Arabidopsis thaliana. *Plant J* **16**, 735-743 (1998).

4 Hiscox, J. D. & Israelstam, G. F. A method for the extraction of chlorophyll from leaf tissue without maceration. *Canadian Journal of Botany* **57**, 1332-1334 (1979).

5 Obayashi, T. & Kinoshita, K. Rank of correlation coefficient as a comparable measure for biological significance of gene coexpression. *DNA research : an international journal for rapid publication of reports on genes and genomes* **16**, 249-260, doi:10.1093/dnares/dsp016 (2009).
